# Genome-Wide Association Meta-Analysis of Single-Nucleotide Polymorphisms and Symptomatic Venous Thromboembolism during Therapy for Acute Lymphoblastic Leukemia and Lymphoma in Caucasian Children

**DOI:** 10.3390/cancers12051285

**Published:** 2020-05-19

**Authors:** Marion K. Mateos, Morten Tulstrup, Michael CJ Quinn, Ruta Tuckuviene, Glenn M. Marshall, Ramneek Gupta, Chelsea Mayoh, Benjamin O. Wolthers, Pasquale M. Barbaro, Ellen Ruud, Rosemary Sutton, Pasi Huttunen, Tamas Revesz, Sonata S. Trakymiene, Draga Barbaric, Ulf Tedgård, Jodie E. Giles, Frank Alvaro, Olafur G. Jonsson, Françoise Mechinaud, Kadri Saks, Daniel Catchpoole, Rishi S. Kotecha, Luciano Dalla-Pozza, Georgia Chenevix-Trench, Toby N. Trahair, Stuart MacGregor, Kjeld Schmiegelow

**Affiliations:** 1 Kids Cancer Centre, Sydney Children’s Hospital Randwick, Sydney, NSW 2031, Australia; Glenn.Marshall@health.nsw.gov.au (G.M.M.); Draga.Barbaric@health.nsw.gov.au (D.B.); Toby.Trahair@health.nsw.gov.au (T.N.T.); 2 School of Women and Children’s Health, University of New South Wales (UNSW), Sydney, NSW 2052, Australia; RSutton@ccia.org.au; 3 Children’s Cancer Institute, Lowy Cancer Research Centre, UNSW, Sydney, NSW 2052, Australia; cmayoh@ccia.org.au (C.M.); JGiles@ccia.org.au (J.E.G.); 4 Department of Pediatrics and Adolescent Medicine, University Hospital Rigshospitalet, 2100 Copenhagen, Denmark; morten.rytter.tulstrup@regionh.dk (M.T.); benjamin.ole.wolthers@regionh.dk (B.O.W.); Kjeld.Schmiegelow@regionh.dk (K.S.); 5 Statistical Genetics Laboratory, QIMR Berghofer Medical Research Institute, Herston, Brisbane, QLD 4006, Australia; Michael.Quinn3@health.qld.gov.au (M.C.J.Q.); Stuart.MacGregor@qimrberghofer.edu.au (S.M.); 6 Department of Pediatrics, Aalborg University Hospital, Hobrovej 18-22, 9000 Aalborg, Denmark; rt@rn.dk; 7 Department of Health Technology, Technical University of Denmark, 2800 Kongens Lyngby, Denmark; ramneek@bioinformatics.dtu.dk; 8 Children’s Medical Research Institute, University of Sydney, Westmead, Sydney, NSW 2145, Australia; Pasquale.Barbaro@health.qld.gov.au; 9 Queensland Children’s Hospital, Brisbane, QLD 4101, Australia; 10 Department of Pediatric Hematology and Oncology, Division of Pediatric and Adolescent Medicine, Oslo University Hospital, 0424 Oslo, Norway; elruud@ous-hf.no; 11 Institute for Clinical Medicine, Faculty of Medicine, University of Oslo, 0318 Oslo, Norway; 12 Department of Pediatric Hematology, Oncology and Stem Cell Transplantation, New Children’s Hospital, Helsinki University Hospital, Stenbäckinkatu 9, 00290 Helsinki, Finland; Pasi.Huttunen@hus.fi; 13 Women’s and Children’s Hospital, North Adelaide, SA 5006, Australia; Tom.Revesz@sa.gov.au; 14 Children’s Hospital, Affiliate of Vilnius University Hospital Santaros Klinikos, Santariškių Str. 7, LT-08406 Vilnius, Lithuania; sonatrak@gmail.com; 15 Department of Pediatric Hematology and Oncology, Skåne University Hospital, Lasarettsgatan 48, 221 85 Lund, Sweden; Ulf.Tedgard@skane.se; 16 Department of Clinical Sciences Lund, Pediatrics, Lund University, Sölvegatan 19, BMC F12 Lund, Sweden; 17 John Hunter Children’s Hospital, Newcastle, NSW 2305, Australia; Frank.Alvaro@hnehealth.nsw.gov.au; 18 School of Medicine and Public Health, University of Newcastle, University Drive Callaghan, Newcastle, NSW 2308, Australia; 19 Children’s Hospital, Barnaspitali Hringsins, Landspitali University Hospital, Hringbraut 101, 101 Reykjavik, Iceland; olafurgi@landspitali.is; 20 The Royal Children’s Hospital, Parkville, Melbourne, VIC 3052, Australia; francoise.mechinaudheloury@aphp.fr; 21 Unite Hematologie Immunologie, Hopital universitaire Robert-Debre, 75019 Paris, France; 22 Department of Hematology and Oncology, Tallinn Children’s Hospital, 13419 Tallinn, Estonia; kadri.urbsoo@mail.ee; 23 Tumour Bank, Children’s Cancer Research Unit, The Children’s Hospital at Westmead, Westmead Sydney, NSW 2145, Australia; daniel.catchpoole@health.nsw.gov.au; 24 Perth Children’s Hospital, Nedlands, Perth, WA 6009, Australia; Rishi.Kotecha@health.wa.gov.au; 25 Telethon Kids Cancer Centre, Telethon Kids Institute, University of Western Australia, Nedlands Perth, WA 6009, Australia; 26 School of Pharmacy and Biomedical Sciences, Curtin University, Bentley, Perth, WA 6102, Australia; 27 Cancer Centre for Children, The Children’s Hospital at Westmead, Westmead, Sydney, NSW 2145, Australia; luciano.dallapozza@health.nsw.gov.au; 28 Children’s Cancer Research Unit, The Children’s Hospital at Westmead, Westmead, Sydney, NSW 2145, Australia; 29 Cancer Genetics Laboratory, QIMR Berghofer Medical Research Institute, Herston, Brisbane, QLD 4006, Australia; Georgia.Trench@qimrberghofer.edu.au; 30 Institute of Clinical Medicine, Faculty of Medicine, University of Copenhagen, 2200 Copenhagen, Denmark

**Keywords:** acute lymphoblastic leukemia, child, genome-wide association study, single-nucleotide polymorphism, venous thromboembolism

## Abstract

Symptomatic venous thromboembolism (VTE) occurs in five percent of children treated for acute lymphoblastic leukemia (ALL), but whether a genetic predisposition exists across different ALL treatment regimens has not been well studied. Methods: We undertook a genome-wide association study (GWAS) meta-analysis for VTE in consecutively treated children in the Nordic/Baltic acute lymphoblastic leukemia 2008 (ALL2008) cohort and the Australian Evaluation of Risk of ALL Treatment-Related Side-Effects (ERASE) cohort. A total of 92 cases and 1481 controls of European ancestry were included. Results: No SNPs reached genome-wide significance (*p* < 5 × 10^−8^) in either cohort. Among the top 34 single-nucleotide polymorphisms (SNPs) (*p* < 1 × 10^−6^), two loci had concordant effects in both cohorts: *ALOX15B* (rs1804772) (MAF: 1%; *p* = 3.95 × 10^−7^) that influences arachidonic acid metabolism and thus platelet aggregation, and *KALRN* (rs570684) (MAF: 1%; *p* = 4.34 × 10^−7^) that has been previously associated with risk of ischemic stroke, atherosclerosis, and early-onset coronary artery disease. Conclusion: This represents the largest GWAS meta-analysis conducted to date associating SNPs to VTE in children and adolescents treated on childhood ALL protocols. Validation of these findings is needed and may then lead to patient stratification for VTE preventive interventions. As VTE hemostasis involves multiple pathways, a more powerful GWAS is needed to detect combination of variants associated with VTE.

## 1. Introduction

Symptomatic venous thromboembolism (VTE) is a well-recognised complication that can lead to substantial morbidity and potential mortality during therapy for childhood acute lymphoblastic leukemia (ALL) [1,2,3,4]. Children and adolescents who experience VTE during ALL treatment are at risk of interruption or discontinuation of chemotherapy, which can lead to decreased survival [5]. Malignancy itself along with treatment-related factors such as steroids, asparaginase, immobilization, and use of central venous lines (CVLs) increase the risk of VTE [6,7]. In addition, older age is a significant risk factor for VTE [2,3,4]. However, it is unknown whether common germline DNA variants play a significant role in development of thrombosis during treatment for ALL [4,8]. There is a paucity of genetic studies, especially a lack of studies based on genome-wide approaches in children with VTE, and particularly in children who share a similar phenotype [4,8,9].

Accordingly, we conducted a genome-wide association study (GWAS) meta-analysis to identify single-nucleotide polymorphisms that were associated with risk of VTE for children and adolescents treated on childhood ALL protocols in two international cohorts.

## 2. Results

### 2.1. Patient Characteristics of Nordic Society of Pediatric Hematology and Oncology (NOPHO) and Australian VTE in ALL Cohorts

**Figure 1:**
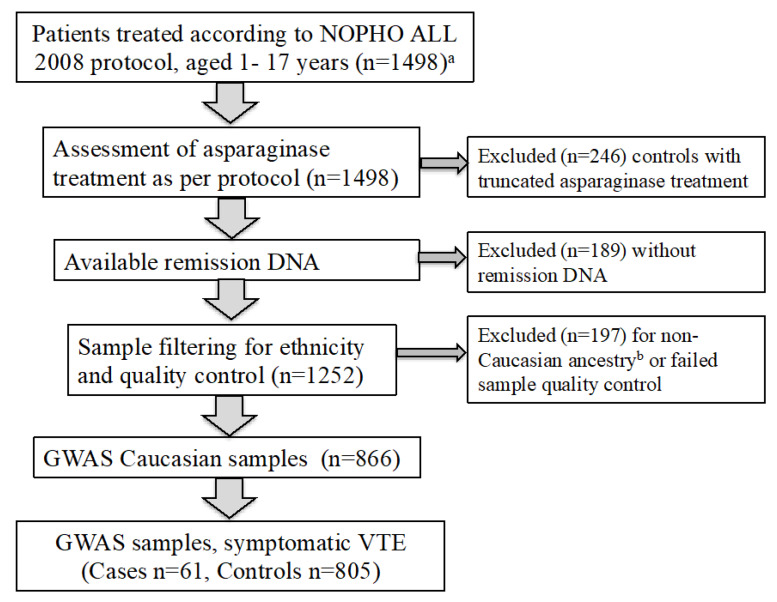
Consort diagram of 866 individuals in the Nordic Society of Pediatric Hematology and Oncology (NOPHO) genome-wide association study (GWAS) discovery cohort for symptomatic venous thromboembolism (VTE). ^a^ NOPHO clinical cohort as described in Rank, et al. 2018 and Tuckuviene, et al. 2016. In addition, there were three individuals who experienced VTE but who did not fulfil all inclusion criteria for the protocol (two mixed-phenotype acute leukemia (MPAL), one pre-treatment). ^b^ Caucasian ethnicity defined by CEU, (Utah Residents (CEPH) with Northern and Western European Ancestry) clustering in principal component analysis (see methods). The final NOPHO GWAS cohort for symptomatic VTE comprised 61 cases (those who experienced symptomatic VTE during first-line ALL treatment) and 805 controls (those who did not experience symptomatic VTE during first-line ALL treatment).

**Figure 2:**
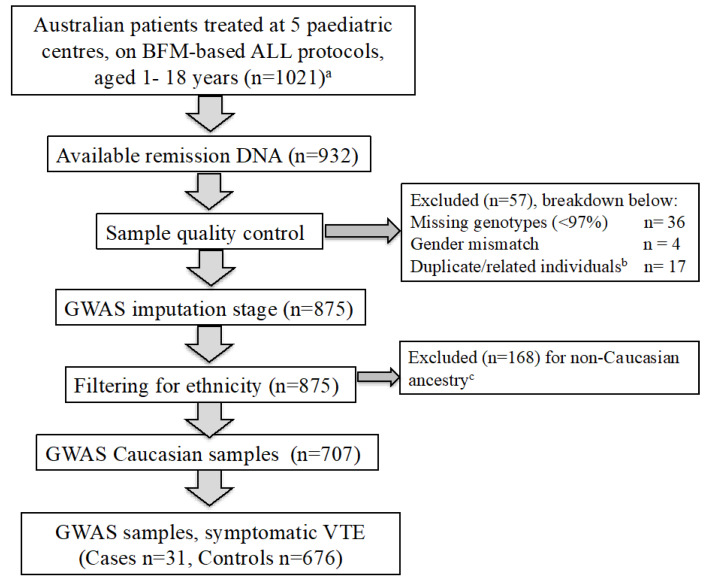
Consort diagram of 707 individuals in the Evaluation of Risk of ALL Treatment-related Side-Effects (ERASE) GWAS discovery cohort for symptomatic VTE. ^a^ ERASE clinical cohort described in Mateos, et al. 2019. ^b^ Pi-hat threshold >0.2. ^c^ Non-Caucasian ancestry defined as per 1000 Genomes data. After quality control and filtering for ethnicity, there were 707 individuals of Caucasian ancestry in the ERASE GWAS analysis.

To study the genetic determinants of symptomatic VTE in acute lymphoblastic leukemia, we analysed data from two published ALL VTE cohorts [2,3,4]. Consort diagrams for patient selection in the GWAS are outlined in Figure 1 and Figure 2 and the baseline demographics of the two cohorts are detailed in Table 1. The cohort consisted of 1573 children and adolescents, aged between 1 and 18 years—92 of whom had experienced VTE at a median time of 80 days from diagnosis of ALL/LBL to occurrence of VTE, while 1481 children did not experience VTE during first-line ALL/LBL treatment. The clinical practice for both cohorts was to site central lines early in treatment. Therefore, this study is not able to study the impact of central line insertion on the incidence of VTE in ALL. There was no routine thromboprophylaxis for patients in either cohort nor replacement of antithrombin III for patients in either cohort.

When analysing time to VTE within each cohort, the ERASE cohort had a median time of onset from cancer diagnosis to VTE diagnosis of 35 days (interquartile range (IQR) 25−164 days, range 1−731 days) while the NOPHO cohort had a median onset from ALL diagnosis to VTE diagnosis of 109 days (IQR 51−137 days, range 24−363 days) (*p* = 0.006).

Most VTE events were deep venous thromboses (*n* = 52, 56.5% of VTE cases), with 31 cases of cerebral venous sinus thrombosis (CVST) (33.7%), and nine cases of pulmonary emboli (PE) (9.8%). Central venous line (CVL)-associated VTE, defined as a VTE occurring in the vein in which a CVL was placed, occurred in a similar proportion of patients in each cohort—46% in the NOPHO2008 compared to 44% in the ERASE cohort [2,4]. All patients listed in Table 1 were included in the respective GWA studies and subsequent meta-analysis (METAL analysis).

### 2.2. METAL Analysis of GWA Studies of NOPHO and Australian VTE Cohorts

The METAL analysis [10] of NOPHO and ERASE ALL cohorts yielded results for 10, 922, 653 single-nucleotide polymorphisms (SNPs), and were ranked according to descending METAL *p* value. To reduce the risk of confounding due to population stratification, each individual cohort analysis and the METAL analysis was performed after exclusion of individuals of non-Caucasian ancestry. No SNPs reached genome-wide association significance (*p* < 5 × 10^−8^). In total, there were 34 SNPs with a *p* value < 10^−6^ (listed in Table 2 and Supplementary Table S1). These corresponded to two loci, taking into account the linkage disequilibrium (LD) of associated SNPs (Table 2). Supplementary Table S1 includes SNPs that had *p* values from the METAL analysis of *p* < 5 × 10^−6^.

**Figure 3:**
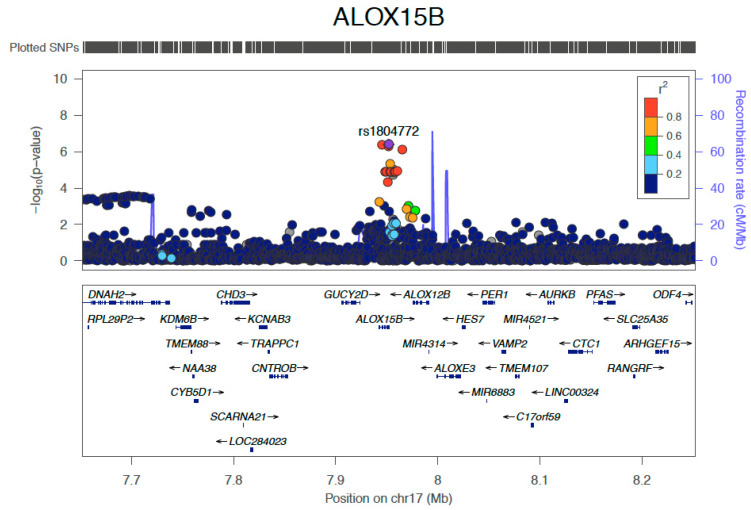
Locus zoom plot of *ALOX15B* top SNP rs1804772. SNPs located either side of rs1804772 (purple dot, labelled), at a distance of 300 kilobases, are shown. r2 refers to linkage disequilibrium with rs1804772, where values closer to 1.0 indicate SNPs in strong linkage disequilibrium with rs1804772.

The most strongly associated SNP in the METAL analysis was rs1804772 (*p* = 3.95 × 10^−7^, minor allele frequency (MAF) 1%, Figure 3) within the *ALOX15B* gene at chromosome 17p13.1. *ALOX15B* codes for arachidonate 15-lipoxygenase type B. The top SNP rs1804772 is found in the 3′ untranslated region of *ALOX15B* and was associated with an increased risk of VTE (OR 8.1 (95%CI 4.6–18.2)).

The MAF of these SNPs in comparison to reference populations is presented in Table 3.

In the NOPHO cohort, heterozygotes (AC) had a 6/28 (21.4%) risk of VTE, whereas homozygotes for the non-risk allele (CC) had a 54/836 (6.5%) risk of VTE. In the ERASE cohort, heterozygotes (AC) had a 6/24 (25%) risk of VTE, and homozygotes CC had a 29/678 (4.3%) risk of VTE. There were no homozygotes for the risk allele. Thus, in the overall cohort, risk of VTE for the AC genotype was 23% and for homozygous CC was 5.4%.

In total, there were three SNPs in *ALOX15B* at *p <* 1 × 10^−6^ that were associated with development of VTE (Table 2 and Supplementary Table S1). The two other SNPs were rs7225107, located in the coding region of *ALOX15B* and rs140958758, which is located close to the 3′end of the transcript. All three SNPs are in LD, where rs1804772 and rs7225107 are in complete LD (D’ = 1, r2 = 1), while rs140958758 is in moderate LD (D’ = 1, r2 = 0.69) [11].

*ALOX15B* has a function in lipid metabolism and arachidonic acid metabolism, and increased expression leads to platelet aggregation in vitro [12]. Thus, variants within the *ALOX15B* gene or regulatory regions are biologically plausible candidates for treatment-related VTE in patients treated for ALL/LBL.

*KALRN*, kalirin RhoGEF kinase, located at 3q21.1, was the second-ranked candidate associated with VTE in our study (top SNP rs570684, *p* = 4.34 × 10^−7^, MAF 1%, OR 0.11 (95%CI 0.05–0.26)). *KALRN* encodes a protein kalirin, with diverse functions including RhoGDP/GTP exchange and inhibition of inducible nitric oxide synthase in neuronal cells [13]. This was the only SNP identified at this locus. Despite a difference in the effect size between the ERASE and NOPHO cohorts (Table 2), we believe that the *KALRN* SNP it is a potential candidate worthy of validation in external other cohorts.

It is relevant to thrombosis that *KALRN* and associated SNPs confer increased risk of ischemic stroke [14], atherosclerosis and early-onset coronary artery disease [15]. 

Within the additional loci that had an association *p* < 5 × 10^−6^ with VTE, we also identified SNP rs140514603 in *RIN3* (Ras and Rab interactor 3, 14q32.12). *RIN3* missense mutations have been found to predispose to Paget’s disease of bone [16]. *RIN3,* specifically rs3742717, was found to be important in a whole-exome study of VTE in childhood ALL [8]. These two SNPs (rs140514603 and rs3742717) are not in LD (D’ = 0.76, r2 = 0.001).

A candidate SNP analysis of the NOPHO cohort identified a significant association between VTE and F11 rs2036914 (hazard ratio (HR) 1.52, 95%CI 1.11–2.07), a borderline significant association with FGG rs2066865 (HR 1.37, 95%CI 0.99–1.91) but no association with either ABO rs8176719 or F5 rs6025 [17]. In contrast, in the ERASE VTE study, no significant associations were found between SNPs associated with VTE in non-cancer populations (Table S2) and VTE in the ERASE cohort [4]. We repeated this analysis using the meta-analysis dataset but did not find any significant association between SNPs associated with VTE in the non-cancer population and VTE in ALL patients (Table S3). The F11 rs2036914 SNP was not in the meta-analysis dataset, but there was no significant association with 4 other F11 SNPs rs925451, rs4253417, rs3756011, rs2289252 (Table S3). We did not reproduce the borderline significant association between VTE and FGG rs2066865 (minor allele frequency 0.25, *p* = 0.66, odds ratio 1.07 (0.76–1.52, 95% confidence interval)) (Table S3). Concordant with the NOPHO analysis we did not identify a significant association between ALL VTE and 21 SNPs in the ABO gene or in linkage disequilibrium with rs8176719 (Table S3). In the meta-analysis dataset, there was no significant association between FVL rs6025 (minor allele frequency 0.02, *p* = 0.36, odds ratio 1.46 (0.64–3.31, 95% confidence interval)), concordant with the NOPHO data [17]. 

## 3. Discussion

To our knowledge, this is the first GWAS meta-analysis that has been reported for childhood ALL/LBL-related symptomatic venous thrombosis. This study was performed after excluding individuals on non-Caucasian ancestry to reduce confounding due to population stratification. Combining two large international cohorts, there were 92 cases and 1481 controls, with calculated 80% power to detect SNPs present at MAF ≥5% with a genotype relative risk of 3.2. Despite this, we did not detect a genome-wide significant candidate. We were not able to demonstrate an association between SNPs associated with VTE in non-cancer populations and VTE occurring in ALL patients. Whilst there are limitations to the power of our meta-analysis, the data suggest that there may be distinct pathophysiologic mechanisms underlying VTE in ALL patients compared to VTE in non-cancer populations. Top candidate SNPs were associated with *ALOX15B* (rs1804772) and *KALRN* (rs570684) (*p* < 1 × 10^−6^). A strength of this study is that the top SNPs were identified despite the heterogeneity of treatment protocols. If validated, these top SNPs could uncover genetic susceptibility mechanisms for VTE. This may assist with identifying a population of children at highest risk of VTE during therapy for ALL/LBL, who may benefit from thromboprophylaxis [7,18].

Several lines of evidence demonstrate that proteins implicated from this GWAS are functionally important for platelet activation (ALOX15B [12]), lipid mobilization/atherosclerosis (ALOX15B [12], kalirin [15]) and endothelial function (kalirin [19]).

The top SNP rs1804772 is associated with gene expression changes in tibial artery tissue, via a linked gene *KRBA2* (Open Targets Platform, https://www.targetvalidation.org, accessed July 17 2019, [20]).

*ALOX15B* and direct metabolic by-products, including 15-hydroxyeicosatetraenoic acid, have a role in atherosclerosis and ischemic stroke in adults [12]. *ALOX15B* is induced following macrophage activation by pro-inflammatory cytokines and lipids [12]. Increased expression of *ALOX15B* induced platelet aggregation and increased thrombin generation that was independent of tissue factor [12] and in vitro modelling in the same study demonstrated that knockdown of *ALOX15B* reversed the thrombogenic phenotype. Pharmacological inhibition of ALOX12/15 in a mouse model of ischemic stroke led to neuronal protection and improved behavioural outcomes [21].

Kalirin, encoded by *KALRN*, is present in extracellular vesicles secreted by TNF-α-activated venous endothelial cells in vitro [19]. *KALRN* expression resulted in inhibition of inducible nitric oxide synthase (iNOS) in pituitary cells in vivo and a possible neuroprotective effect [13]; thus further study of kalirin and iNOS induction in endothelial cells may be important.

If validated, the top SNPs may reveal new avenues for further study in ALL/LBL-related VTE. One testable hypothesis is that lipid and cytokine-induced inflammation during ALL therapy leads to platelet activation, venous endothelial dysfunction and altered thrombotic risk. This inflammatory process could be altered by genetic polymorphisms (such as those identified in this study), chemotherapy [6,22] and/or other environmental factors such as infection [3,4]. Supporting evidence includes that asparaginase and steroids act synergistically to increase circulating serum lipids and triglycerides [22,23,24] with a possible increased thrombotic risk [22]; and that platelets are known to express receptors for cytokines and oxidised lipids on their cell membrane, such as CD36 [25]. Thus, through improved understanding of thrombogenic mechanisms in ALL/LBL, there may be potential to exploit tissue and endothelial protection.

Results of the current study and a recent whole-exome study [8] also warrant further study of the function of *RIN3* in ALL/LBL treatment-related VTE.

This study highlights challenges and limitations in conducting a GWAS to investigate ALL treatment-related VTE. Large cohort studies are required to provide sufficient power to detect variants associated with rare conditions. This is in order to refute the null hypothesis, that there are no SNPs associated with the phenotype at GWS levels (*p* < 5 × 10^−8^). The GWAS meta-analysis was underpowered to detect SNPs with MAF <5% and genotype relative risk <3.2 [26]. To detect SNPs with MAF 5% and associated genotype relative risk of 2, with 80% power, 444 cases were required. This also demonstrates that in order to detect SNPs with a weaker effect on the phenotype, very large cohorts are required. Secondly, most GWA studies are conducted in homogenous ethnic populations, to reduce risk of population stratification [27]. As this GWAS was conducted in children and adolescents of Caucasian ancestry, results cannot be extrapolated to non-Caucasian populations. The incidence of VTE between the NOPHO2008 and ERASE cohorts was 6.1% and 5.1% respectively [2,4]. The meta-analysis, as outlined in the patients and methods and consort diagrams (Figure 1 and Figure 2), includes the subset of NOPHO and ERASE patients with available DNA samples, Caucasian ancestry and where the sample passed quality control. As a result of sample availability and filtering, 61/63 NOPHO VTE cases were included in the meta-analysis. In contrast, there was a lower proportion of ERASE VTE cases included in meta-analysis (31/52) due to the filtering criteria. Finally, there was heterogeneity in the timing of VTE events between the ERASE and NOPHO cohorts, likely due to asparaginase scheduling, with VTE events occurring significantly earlier in the ERASE cohort. For NOPHO2008, patients received asparaginase therapy from week 4 to week 36, during the consolidation, delayed intensification and maintenance phases of treatment [2]. In contrast, patients in the ERASE cohort received asparaginase therapy during induction therapy (weeks 1–5) and re-induction therapy (weeks 24–28) [4]. This limitation will be difficult to overcome in larger international GWA studies, due to variation in ALL treatment protocols worldwide.

## 4. Patients and Methods

We conducted two separate genome-wide association studies—one in a Nordic Society of Pediatric Hematology and Oncology (NOPHO) cohort comprising 61 cases and 805 controls, and one in an Australian cohort (ERASE), comprising 31 cases and 676 controls. Cases were defined as patients who had experienced a symptomatic VTE (termed “VTE” in this study) during first-line ALL therapy, while controls had not experienced a symptomatic VTE. 

*NOPHO GWAS:* All included patients were diagnosed with B-cell precursor, T-cell ALL or mixed-phenotype acute leukemia (MPAL) between 2008 and 2017 and were treated according to the NOPHO ALL2008 protocol. Patients were aged between 1 and 17 years inclusive. This protocol and the occurrence of thromboembolism in the cohort have been described in detail elsewhere [2,3]. All patients with Down syndrome were excluded. Included cases had all experienced a symptomatic VTE, according to the Ponte di Legno Toxicity Working Group (PTWG) severity grade 2A or higher [28]. We excluded all controls that did not complete their full PEG-asparaginase therapy as prescribed in the protocol. Asparaginase is used from consolidation onwards in the NOPHO ALL2008 protocol, and anticipated asparagine depletion is from Week 5 to Week 35 [29].

Genotyping was performed on the Illumina Infinium Omni 2.5exome-8-BeadChip using DNA from remission blood samples. We removed SNPs with a genotype call rate <98%, a minor allele frequency (MAF) <1%, or with evidence of Hardy–Weinberg deviation (*p* < 5 × 10^−6^). Samples were filtered based on individual missingness (>2%), excess heterozygosity (±4 standard deviations (SD)), mismatch between sex according to genotype and clinical data, and related individuals (as determined by identity-by-state estimates). Genetic ancestry was determined by multidimensional scaling analysis including HapMap reference samples and excluded all patients >15 SD from the European (CEU, Utah Residents (CEPH) with Northern and Western European Ancestry) cluster mean. The NOPHO cohort consort diagram is shown in Figure 1. Association analyses based on 1,446,007 available SNPs were conducted using logistic regression with adjustment for age, sex, risk group, and genetic ancestry as determined by principal components analysis.

*Evaluation of ALL Treatment-related Side-Effects (ERASE) GWAS:* Included patients were diagnosed with ALL or lymphoblastic lymphoma (LBL) between 1998 and 2013 in Australia, aged between 1 and 18 years inclusive, and treated on one of the following BFM-based protocols: ANZCCSG study 7, ANZCHOG study 8, BFM-95, COG A5971, or AIEOP-BFM study 9 [30,31,32,33,34]. LBL patients were included, as they were treated on the same protocols as patients diagnosed with ALL. The ERASE study has been described in detail previously [4]. In contrast to NOPHO ALL2008, asparaginase is used in the induction phase in BFM-based protocols. Patients were excluded as controls if they had less than 18 months of follow up after diagnosis. Thromboembolic events were classified according to the Common Terminology Criteria for Adverse Events (CTCAE) [35], and all grades of symptomatic events were included as cases.

Germline DNA was genotyped on the Illumina Infinium Oncoarray-530K BeadChip. Samples were filtered by individual missingness (>3%), sex mismatch, and related individuals. We removed SNPs with a genotype call rate < 97%, MAF < 1%, and Hardy–Weinberg *p* value < 10^−4^. The association analyses were restricted to patients of Caucasian ancestry, as defined by <6 SD from the following groups in the 1000 Genomes reference panel [36]: IBS (Iberian population in Spain), TSI (Toscani in Italy), GBR (British in England and Scotland), CEPH (Utah residents with Northern and Western European ancestry), and FIN (Finnish in Finland). The ERASE cohort consort diagram is shown in Figure 2. After quality control, imputation analysis (reference panel 1000 Genomes European) was performed using IMPUTE2 [37] and the analysis excluded imputed SNPs with an information score <0.4, resulting in 10, 922, 652 SNPs available for analysis. The GWAS was performed as a logistic regression with adjustment for age, sex and genetic ancestry as determined by principal component analysis.

*Meta-analysis*: After the two separate GWA studies, we carried out a meta-analysis in METAL, using the Standard Error SE model (STDERR) which takes into account effect size estimates and standard errors [10].

With 80% power, the GWA meta-analysis could detect VTE-associated SNPs of a MAF ≥ 6% with a relative risk (RR) of 3.2 for the minor allele; or a MAF ≥ 5% and a RR of 3.5; or a MAF of 3–4% and a RR at or above 4.0 [26].

*Top SNPs*: For SNPs in the GWAS meta-analysis, the following data were reviewed: *P* value, minor allele frequencies, odds ratio (OR), OR 95% confidence interval, annotated gene, and genomic position. The top SNPs were those that had *p* values from the METAL analysis of *p* < 5 × 10^−6^. The annotated gene was determined by cross-referencing available online genome browsers Refseq [38], ensembl 74 [39] and UCSC database information (GRCh37/hg19, 2015 update [40]) accessed through SNPnexus (2012 update, [41]). The SNPnexus database (http://www.snp-nexus.org) is kept synchronised with the UCSC human genome annotation database (http://genome.ucsc.edu). Where there was discrepancy or the gene was uncertain, a search was performed manually using NCBI dbSNP build 151 [42]. Linkage disequilibrium (LD) of candidate SNPs was examined using the LDlink online tool (https://ldlink.nci.nih.gov) [11].

We had previously compiled a list of germline SNPs associated with VTE in the non-cancer population based on several adult series, one pediatric study and SNPs identified through dbSNP build 149 (Supplementary Table S2) [9,43,44,45,46,47,48,49]. The list of SNPs which was expanded to include additional SNPs in complete linkage disequilibrium (r2 = 1), using the SNP annotation and proxy (SNAP) search tool with HapMap genomic data [50].

*Ethics statement*: This study and database were approved by the relevant local and national ethical bodies from Denmark (for NOPHO, approval H-2.2010-002) and Australia (for the ERASE Study). The ERASE study was approved by the Hunter New England Human Research Ethics Committee (HNEHREC Reference Number: 12/11/21/4.01).

## 5. Conclusions

In summary, we have identified biologically plausible candidate SNPs that associate with risk of VTE in childhood ALL therapy across two international cohorts which require validation in future studies. A large international GWAS with capacity for discovery and validation cohorts would help advance the field of understanding in childhood ALL/LBL-related thrombosis. Such a study is currently being designed within the PTWG. A genotype risk signature could also be considered to assess cumulative risk with combinations of risk alleles [17]. A reduction in phenotypic variation, by selection of a VTE subtype such as CVST, may also increase the strength of association and risk effect exerted by candidate SNPs.

## Figures and Tables

**Table 1:** Demographics of children and adolescents experiencing VTE * Age is rounded to closest year in the ERASE cohort, as initial age values were in months. ^†^ Other = lineage not known, includes mixed-phenotype acute leukemia (*n* = 7 in ERASE cohort, *n* = 2 in NOPHO cohort), ALL/LBL (lineage not specified). For comparison of NOPHO and ERASE cohorts, two-sided *p* values < 0.05 were considered significant; NC = *p* value was not calculated. Abbreviations: IQR, interquartile range; ALL, acute lymphoblastic leukemia; LBL, lymphoblastic lymphoma; T21, Trisomy 21; Ph + ALL, Philadelphia chromosome (t(9;22) positive ALL; CVST, cerebral venous sinus thrombosis; DVT, deep venous thrombosis; PE, pulmonary embolus. ANZCCSG, Australian and New Zealand Children’s Cancer Study Group; ANZCHOG, Australian and New Zealand Children’s Hematology and Oncology Group; AIEOP, Associazone Italiana Ematologia Oncologia Pediatrica; BFM, Berlin-Frankfurt-Münster; COG, Children’s Oncology Group; NOPHO, Nordic Society of Pediatric Hematology and Oncology.

Clinical Feature	Category	GWAS Cohort (Total *n* = 1573)		*p* Value
		ERASE	NOPHO	
VTE	Yes	31	61	0.033
	No	676	805	
Age at diagnosis (years *)	Median (range)	5 (1−18)	4 (1−17)	0.75
	IQR	3–9	2–8	
Sex	Female	337	394	0.419
	Male	370	472	
Diagnosis	B-ALL	620	752	NC
	B-LBL	4	0	
	T-ALL	60	112	
	ALL/LBL other ^†^	15	2	
	T-LBL	8	0	
Cytogenetics	*ETV6*-*RUNX1* fusion	76	194	NC
	High hyperdiploidy	191	260	
	Hypodiploid	10	10	
	*KMT2A (MLL)* gene rearrangement	12	0	
	Normal	179	150	
	Ph + ALL	5	0	
	Cytogenetics—no result	68	69	
	Cytogenetics—other	150	183	
	Constitutional T21	16	0	
VTE location	CVST	9	22	0.184
	DVT	21	31	
	PE	1	8	
Study protocol	ANZCCSG study 7	193	NOPHO ALL2008 *n* = 866	NC
	ANZCHOG study 8	427		
	BFM 95	59		
	COG A5971	2		
	AIEOP-BFM study 9	26		

**Table 2:** Top single-nucleotide polymorphisms (SNPs) in VTE meta-analysis, *p* < 1 × 10^−6^. Top SNPs associated with risk of VTE in ALL/LBL (note only top SNP per loci (lowest *p* value) included, significance level *p* < 1 × 10^−6^). “Chr”, chromosome; “Position”, genomic position; “SNP ID”, rs identification number for single-nucleotide polymorphism; “Allele 1” and “Allele 2” refer to nomenclature from METAL meta-analysis software, where Allele 1 is the Reference allele; “MAF”, minor allele frequency, as per Genomic Coordinates in SNPNexus database (http://www.snp-nexus.org) which is synchronised with the UCSC human genome annotation database (http://genome.ucsc.edu); “StdError”, standard error; “Direction” refers to direction of effect of the SNP in each cohort, thus “++” refers to concordant positively-associated effect with VTE risk. SNPs were excluded from this table if the SNP was only able to be analysed in one cohort within the meta-analysis. “OR”, odds ratio; “Lower 95C”, lower 95% confidence interval of OR; “Upper 95%CI”, upper 95% confidence interval of OR; “Gene” and “location” refer to consensus gene and location of the SNP in relation to introns/exons of the associated gene, determined through SNPnexus cross-referencing of UCSC, Refseq and Ensembl databases. Where there was discordance, the information from dbSNP 151 was used. “Intronic”, located in the intron of a coding gene; “3utr”, located within 3 prime untranslated region.

Chr	Position	SNP ID	Gene	Location	Allele		MAF	Cohort	Effect	Std Err	*p* value	Direction	OR	Lower 95%CI	Upper 95%CI
					1	2									
chr17	8,048,687	rs1804772	*ALOX15B*	3utr	a	c	0.01	Combined	2.09	0.41	3.95 × 10^−7^	++	8.1	3.61	18.18
								ERASE	2.8	0.64	9.38 × 10^−5^				
								NOPHO	2.1	0.55	1.38 × 10^−4^				
chr3	124,403,999	rs570684	*KALRN*	intronic	t	c	0.01	Combined	−2.18	0.43	4.34 × 10^−7^	--	0.11	0.05	0.26
								ERASE	−17.85	18.46	8.93 × 10^−2^				
								NOPHO	−2	0.46	1.17 × 10^−5^				

**Table 3:** Minor allele frequencies for *ALOX15B* and *KALRN* SNPs in reference populations.

Study/Population	*ALOX15B*				*KALRN*
	rs1804772 (3′ Prime UTR Variant) C > A	rs73972650 (Intron Variant) A > G	rs7225107 (Coding) A > G,T	rs140958758 (3′ Downstream) A > C	rs570684 (Intron Variant) T > A,C
This meta-analysis	0.01	0.01	0.01	0.01	0.01
Northern Sweden	0.007	0.007	0.007	0.007	0.035
TWINSUK	0.008	0.008	0.008	0.007	0.007
ALSPAC	0.010	0.010	0.010	0.008	0.004
Estonian	0.018	0.018	0.018	0.018	0.015
GnomAD	0.079	0.074	0.080	0.020	0.089
TOPMED	0.083	0.076	0.083	0.017	0.096
1000Genomes	0.106	0.100	0.107	0.037	0.128

Minor allele frequency as reported in dbSNP, available online at https://www.ncbi.nlm.nih.gov/snp/. NorthernSweden from the Northern Sweden Population Health Study, TWINSUK from the TwinsUK registry study, ALSPAC from the Avon Longitudinal Study of Parents and Children, Estonian from the Estonian Biocentre, GnomAD from The Genome Aggregation Database, TOPMED from the Trans-Omics for Precision Medicine (TOPMed) Program of the NIH National Heart, Lung and Blood Institute and 1000 Genomes from the International Genome Sample Resource and 1000 Genomes project.

## Supplementary Materials

The following are available online at https://www.mdpi.com/2072-6694/12/5/1285/s1, Table S1: SNPs associated with VTE in meta-analysis, *p <* 5 × 10^−6^, Table S2: SNPs associated with VTE in the non-cancer population, Table S3: Lookup table of SNPs associated with VTE in non cancer patients in this meta-analysis.

## Abbreviations

ALL	Acute lymphoblastic leukemia;
ANZCCSG	Australian and New Zealand Children’s Cancer Study Group;
ANZCHOG	Australian and New Zealand Children’s Hematology and Oncology Group;
BFM	Berlin-Frankfurt-Münster;
CI	Confidence interval;
COG	Children’s Oncology Group;
CVL	Central venous line;
CVST	Cerebral venous sinus thrombosis;
DVT	Deep venous thrombosis;
ERASE	Evaluation of Risk of ALL Treatment-Related Side-Effects;
GWAS	Genome-wide association study;
GWS	Genome-wide significance;
LBL	Lymphoblastic lymphoma;
LD	Linkage disequilibrium;
MAF	Minor allele frequency;
MPAL	Mixed-phenotype acute leukemia;
NOPHO	Nordic Society of Pediatric Hematology and Oncology;
NCI-CTCAE	National Cancer Institute Common Terminology Criteria for Adverse Events;
OR	Odds ratio;
SD	Standard deviations;
SNP	Single-nucleotide polymorphism;
PE	Pulmonary embolus;
VTE	Symptomatic venous thromboembolism.
