# Supplementary material for: Genome-Wide Association Meta-Analysis of Single-Nucleotide Polymorphisms and Symptomatic Venous Thromboembolism during Therapy for Acute Lymphoblastic Leukemia and Lymphoma in Caucasian Children
Source: Cancers (Basel). 2020 May 19;12(5):1285. doi: 10.3390/cancers12051285 (PMC7280960; doi:10.3390/cancers12051285)
Supplement: Supplementary file 1 [file cancers-12-01285-s001.pdf]

Table S1

Table S1: SNPs associated with VTE in meta-analysis, $P<5x10^{-6}$ (excluding SNPs in Table 2) One hundred and fourteen SNPs were associated with risk of VTE in ALL, significance level P $<5x10^{-6}$, excluding top SNPs listed in Table 2. SNPs are ordered by chromosomal position. "Chr", chromosome, "Position", genomic position, "SNP ID", rs identification number for single nucleotide polymorphism. "Allele 1" and "Allele 2" refer to nomenclature from METAL meta-analysis software, where Allele 1 is the Reference allele. "MAF", minor allele frequency, as per Genomic Coordinates in SNPNexus database (http://www.snp-nexus.org) which is synchronised with the UCSC human genome annotation database (http://genome.ucsc.edu). "StdError", standard error. "Direction" refers to direction of effect of the SNP in each cohort, thus "++" refers to concordant positively- associated effect with VTE risk, "-?" refers to a negative effect in the first cohort, and an unknown effect in the second cohort. "OR", odds ratio. "Lower_95C", lower 95% confidence interval of OR, "Upper_95CI", upper 95% confidence interval of OR. "Gene" and "location" refers to consensus gene and location of the SNP in relation to introns/exons of the associated gene, determined through SNPnexus cross-referencing of UCSC, Refseq and Ensembl databases. Where there was discordance, the information from dbSNP 151 was used. "Intronic", located in the intron of a coding gene, "3utr", located within 3 prime untranslated region, "coding" located in the exonic region of a protein coding gene, "non-coding intronic", located in intron of a non-coding gene, "3downstream", located 2 kb downstream of the 3' end of a transcript. "NA", not applicable or not known.

Chr	Position	SNP ID	Allele 1	Allele 2	MAF	Effect	StdErr	P value	Direction	OR	lower_95CI	upper_95CI	Gene	Location
chr1	73916690	rs138922423	t	ta	0.02	2.77	0.6	$3.16x10^{-6}$	+?	16.02	4.99	51.46	NA	NA
chr1	152001324	rs4845725	a	g	0.04	1.33	0.29	$4.29x10^{-6}$	++	3.8	2.15	6.71	*LOC105371441*	non-coding intronic
chr1	200705097	rs115456075	a	t	0.03	2.14	0.46	$3.23x10^{-6}$	+?	8.51	3.45	20.95	NA	NA
chr3	134066333	rs4546143	t	c	0.39	0.87	0.18	$2.12x10^{-6}$	++	2.39	1.67	3.43	*LOC105374117*	non-coding intronic
chr4	13130547	rs12647774	t	c	0.05	-1.25	0.26	$2.07x10^{-6}$	--	0.29	0.17	0.48	NA	NA
chr4	13138969	rs60440889	t	c	0.05	1.27	0.27	$1.79x10^{-6}$	++	3.55	2.11	5.96	NA	NA
chr4	79882154	rs4356865	a	g	0.12	1.54	0.33	$3.40x10^{-6}$	+?	4.66	2.43	8.91	NA	NA
chr4	79931672	rs10433910	t	c	0.05	-1.95	0.38	$3.76x10^{-7}$	-?	0.14	0.07	0.3	*ANTXR2*	intronic
chr4	79935918	rs12647878	a	g	0.05	-1.95	0.38	$3.58x10^{-7}$	-?	0.14	0.07	0.3	*ANTXR2*	intronic
chr4	79937183	rs4690128	a	t	0.05	-1.95	0.38	$3.59x10^{-7}$	-?	0.14	0.07	0.3	*ANTXR2*	intronic
chr4	79950123	rs34387116	a	aat	0.05	1.95	0.38	$3.57x10^{-7}$	+?	7.01	3.31	14.82	*ANTXR2*	intronic
chr4	79954323	rs12643472	t	g	0.05	-1.94	0.38	$3.47x10^{-7}$	-?	0.14	0.07	0.3	*ANTXR2*	intronic
chr4	79955754	rs62298605	t	c	0.05	1.94	0.38	$3.45x10^{-7}$	+?	6.99	3.31	14.75	*ANTXR2*	intronic
chr4	79956838	rs62298606	a	c	0.05	-1.94	0.38	$3.45x10^{-7}$	-?	0.14	0.07	0.3	*ANTXR2*	intronic
chr4	79958160	rs62298607	a	g	0.05	1.94	0.38	$3.43x10^{-7}$	+?	6.98	3.31	14.73	*ANTXR2*	intronic
chr4	79973937	rs12645327	a	g	0.05	1.94	0.38	$3.22x10^{-7}$	+?	6.97	3.31	14.66	*ANTXR2*	intronic
chr4	79985869	rs62298610	t	g	0.05	-1.94	0.38	$3.14x10^{-7}$	-?	0.14	0.07	0.3	*ANTXR2*	intronic

Chr	Position	SNP ID	Allele 1	Allele 2	MAF	Effect	StdErr	P value	Direction	OR	lower_95CI	upper_95CI	Gene	Location
chr4	79986490	rs62298611	t	g	0.05	1.94	0.38	3.14×10^{-7}	+?	6.97	3.31	14.66	*ANTXR2*	intronic
chr4	79986779	rs4690131	a	c	0.05	-1.94	0.38	3.14×10^{-7}	-?	0.14	0.07	0.3	*ANTXR2*	intronic
chr4	79987144	rs62298612	t	c	0.05	-1.94	0.38	3.14×10^{-7}	-?	0.14	0.07	0.3	*ANTXR2*	intronic
chr4	79993908	rs60195747	a	g	0.05	-1.94	0.38	3.11×10^{-7}	-?	0.14	0.07	0.3	*ANTXR2*	intronic
chr4	79997830	rs62298615	a	g	0.05	1.94	0.38	3.08×10^{-7}	+?	6.97	3.31	14.66	*ANTXR2*	intronic
chr4	80001133	rs4690133	t	c	0.05	-1.94	0.38	3.06×10^{-7}	-?	0.14	0.07	0.3	*ANTXR2*	intronic
chr4	80012071	rs7682733	a	g	0.05	-1.18	0.23	2.90×10^{-7}	--	0.31	0.2	0.48	*ANTXR2*	intronic
chr4	80015987	rs4690136	a	g	0.05	1.95	0.38	2.99×10^{-7}	+?	7.05	3.34	14.88	*ANTXR2*	intronic
chr4	80020585	rs12646448	a	g	0.05	1.93	0.38	3.37×10^{-7}	+?	6.92	3.29	14.55	*ANTXR2*	intronic
chr4	80023726	rs12643227	t	c	0.05	1.93	0.38	3.37×10^{-7}	+?	6.92	3.29	14.56	*ANTXR2*	intronic
chr4	80026445	rs62298636	t	c	0.05	1.93	0.38	3.39×10^{-7}	+?	6.92	3.29	14.56	*ANTXR2*	intronic
chr4	80029093	rs62298638	a	t	0.05	-1.92	0.38	4.07×10^{-7}	-?	0.15	0.07	0.31	*ANTXR2*	intronic
chr4	80029791	rs7684375	a	c	0.05	-1.92	0.38	4.42×10^{-7}	-?	0.15	0.07	0.31	*ANTXR2*	intronic
chr4	80029921	rs7689594	t	c	0.05	-1.17	0.23	3.60×10^{-7}	--	0.31	0.2	0.49	*ANTXR2*	intronic
chr4	80030211	rs7689762	a	c	0.05	-1.92	0.38	4.65×10^{-7}	-?	0.15	0.07	0.31	*ANTXR2*	intronic
chr4	80033170	rs62298639	c	g	0.05	1.9	0.38	5.63×10^{-7}	+?	6.72	3.18	14.16	*ANTXR2*	intronic
chr4	80035111	rs6534708	t	c	0.06	1.87	0.38	8.50×10^{-7}	+?	6.5	3.08	13.69	*ANTXR2*	intronic
chr4	80035425	rs62298640	a	c	0.06	1.87	0.38	8.81×10^{-7}	+?	6.48	3.08	13.66	*ANTXR2*	intronic
chr4	80036558	rs75688448	t	g	0.06	-1.86	0.38	1.02×10^{-6}	-?	0.16	0.07	0.33	*ANTXR2*	intronic

Chr	Position	SNP ID	Allele 1	Allele 2	MAF	Effect	StdErr	P value	Direction	OR	lower_95CI	upper_95CI	Gene	Location
chr4	80039238	rs200463121	a	ag	0.06	-1.88	0.39	1.17×10^{-6}	-?	0.15	0.07	0.33	*ANTXR2*	intronic
chr4	80039239	rs35015205	g	gt	NA	-1.88	0.39	1.17×10^{-6}	-?	0.15	0.07	0.33	*ANTXR2*	intronic
chr4	80041695	rs12643343	a	g	0.06	1.84	0.38	1.33×10^{-6}	+?	6.3	2.99	13.28	*ANTXR2*	intronic
chr4	80043223	rs7693864	a	g	0.06	-1.87	0.39	1.91×10^{-6}	-?	0.15	0.07	0.33	*ANTXR2*	intronic
chr4	129240194	rs7677291	t	c	0.34	0.75	0.16	4.45×10^{-6}	++	2.11	1.53	2.9	NA	NA
chr4	149268514	rs56895547	t	g	0.01	3.55	0.74	1.42×10^{-6}	+?	34.65	8.2	146.41	*LINC02355*	non-coding intronic
chr4	149268875	rs72951779	a	g	0.01	3.6	0.74	1.28×10^{-6}	+?	36.56	8.52	156.83	*LINC02355*	non-coding intronic
chr4	149268992	rs56171108	t	c	0.01	3.6	0.74	1.27×10^{-6}	+?	36.47	8.51	156.27	*LINC02355*	non-coding intronic
chr4	149273483	rs72951787	a	t	0.01	3.55	0.74	1.41×10^{-6}	+?	34.67	8.21	146.47	*LINC02355*	non-coding intronic
chr4	149273555	rs72951788	t	c	0.01	-3.55	0.74	1.41×10^{-6}	-?	0.03	0.01	0.12	*LINC02355*	non-coding intronic
chr4	149275338	rs78316366	t	c	0.01	-3.6	0.74	1.26×10^{-6}	-?	0.03	0.01	0.12	*LINC02355*	non-coding intronic
chr4	149284129	rs17026111	c	g	0.01	-3.58	0.74	1.25×10^{-6}	-?	0.03	0.01	0.12	NA	NA
chr4	149284962	rs72951795	a	g	0.01	-3.61	0.74	1.25×10^{-6}	-?	0.03	0.01	0.12	NA	NA
chr4	149285924	rs72951801	a	g	0.01	-3.61	0.74	1.24×10^{-6}	-?	0.03	0.01	0.12	NA	NA
chr4	149286423	rs73859724	a	g	0.01	3.55	0.74	1.40×10^{-6}	+?	34.98	8.26	148.2	NA	NA
chr4	149287204	rs56208724	a	g	0.01	3.61	0.74	1.25×10^{-6}	+?	36.86	8.57	158.48	NA	NA
chr4	149288002	rs7678170	t	g	0.01	3.56	0.74	1.41×10^{-6}	+?	35.05	8.26	148.74	NA	NA

Chr	Position	SNP ID	Allele 1	Allele 2	MAF	Effect	StdErr	P value	Direction	OR	lower_95CI	upper_95CI	Gene	Location
chr4	149288776	rs59242740	t	c	0.01	3.61	0.75	$1.28x10^{-6}$	+?	36.9	8.57	158.95	NA	NA
chr4	149289236	rs2164997	t	g	0.01	-3.61	0.74	$1.30x10^{-6}$	-?	0.03	0.01	0.12	NA	NA
chr4	149291013	rs58022888	t	c	0.01	-3.5	0.73	$1.67x10^{-6}$	-?	0.03	0.01	0.13	NA	NA
chr5	118430038	rs139018151	t	c	0.01	-2.95	0.62	$2.24x10^{-6}$	-?	0.05	0.02	0.18	*LINC02208*	non-coding intronic
chr5	121871526	rs75860696	c	g	0.03	2.64	0.56	$2.92x10^{-6}$	+?	14.02	4.64	42.41	*LOC105379148/ LOC105379149*	non-coding intronic
chr5	121875688	rs140855699	a	c	0.03	2.64	0.56	$2.90x10^{-6}$	+?	14.03	4.64	42.43	*LOC105379149*	non-coding intronic
chr5	121876206	rs75308562	t	c	0.03	-2.64	0.56	$2.89x10^{-6}$	-?	0.07	0.02	0.22	*LOC105379149*	non-coding intronic
chr5	121877132	rs7722758	a	g	0.04	2.59	0.55	$2.85x10^{-6}$	+?	13.3	4.5	39.31	*LOC105379149*	non-coding intronic
chr5	121881493	rs74517758	a	c	0.03	-2.64	0.56	$2.86x10^{-6}$	-?	0.07	0.02	0.22	NA	NA
chr5	121927889	rs79055704	a	t	NA	3.25	0.68	$1.53x10^{-6}$	+?	25.8	6.86	97.11	*NA*	NA
chr6	51658941	rs115072237	t	c	0.02	-1.78	0.38	$2.70x10^{-6}$	--	0.17	0.08	0.36	*PKHD1*	intronic
chr6	92640153	rs72926690	t	c	0.03	1.5	0.32	$3.30x10^{-6}$	++	4.46	2.38	8.38	NA	NA
chr7	130403724	rs73152871	a	g	0.03	-2.64	0.55	$1.64x10^{-6}$	-?	0.07	0.02	0.21	*CEP41*	intronic
chr7	130405922	rs73152874	a	g	0.03	2.42	0.52	$3.65x10^{-6}$	+?	11.29	4.05	31.49	*CEP41*	intronic
chr7	130406417	rs149445855	t	c	0.03	2.42	0.52	$3.65x10^{-6}$	+?	11.29	4.05	31.5	*CEP41*	intronic

Chr	Position	SNP ID	Allele 1	Allele 2	MAF	Effect	StdErr	P value	Direction	OR	lower_95CI	upper_95CI	Gene	Location
chr7	130418259	rs73152876	t	c	0.03	-2.42	0.52	$3.71x10^{-6}$	-?	0.09	0.03	0.25	*CEP41*	intronic
chr7	130420215	rs56283021	a	g	0.03	-2.42	0.52	$3.70x10^{-6}$	-?	0.09	0.03	0.25	*CEP41*	intronic
chr7	130422684	rs73152877	t	c	0.03	2.42	0.52	$3.72x10^{-6}$	+?	11.27	4.04	31.44	*CEP41*	intronic
chr7	130427827	rs73152881	t	c	0.03	2.42	0.52	$3.72x10^{-6}$	+?	11.27	4.04	31.46	*CEP41*	intronic
chr7	130428331	rs146064670	a	g	0.03	2.42	0.52	$3.72x10^{-6}$	+?	11.28	4.04	31.48	*CEP41*	intronic
chr7	130428688	rs189608404	t	c	0.03	-2.42	0.52	$3.72x10^{-6}$	-?	0.09	0.03	0.25	*CEP41*	intronic
chr7	130428922	rs55965115	a	t	0.03	2.54	0.55	$3.15x10^{-6}$	+?	12.69	4.36	36.95	*CEP41*	intronic
chr7	130429508	rs73152882	t	g	0.03	-2.42	0.52	$3.72x10^{-6}$	-?	0.09	0.03	0.25	*CEP41*	intronic
chr7	130430054	rs73152883	a	t	0.03	-2.42	0.52	$3.72x10^{-6}$	-?	0.09	0.03	0.25	*CEP41*	intronic
chr7	130431738	rs73152886	a	g	0.03	-2.42	0.52	$3.71x10^{-6}$	-?	0.09	0.03	0.25	*CEP41*	intronic
chr7	130431996	rs73152887	c	g	0.03	-2.42	0.52	$3.72x10^{-6}$	-?	0.09	0.03	0.25	*CEP41*	intronic
chr7	130432554	rs116899144	t	c	0.03	2.42	0.52	$3.72x10^{-6}$	+?	11.28	4.04	31.5	*CEP41*	intronic
chr7	130432937	rs73152891	a	c	0.03	-2.42	0.52	$3.71x10^{-6}$	-?	0.09	0.03	0.25	*CEP41*	intronic
chr7	130433082	rs73152892	a	g	0.03	2.42	0.52	$3.72x10^{-6}$	+?	11.29	4.04	31.51	*CEP41*	intronic
chr7	130433960	rs55660992	a	g	0.03	2.42	0.52	$3.72x10^{-6}$	+?	11.29	4.04	31.52	*CEP41*	intronic
chr8	102029950	rs16868899	a	g	0.05	1.83	0.4	$3.93x10^{-6}$	+?	6.24	2.87	13.57	*NCALD*	intronic

Chr	Position	SNP ID	Allele 1	Allele 2	MAF	Effect	StdErr	P value	Direction	OR	lower_95CI	upper_95CI	Gene	Location
chr9	5731974	rs117233659	a	c	0.01	-2.22	0.48	$3.75x10^{-6}$	--	0.11	0.04	0.28	*RIC1*	intronic
chr9	21845309	rs67134687	a	g	0.07	-1.99	0.43	$4.18x10^{-6}$	-?	0.14	0.06	0.32	*MTAP*	intronic
chr9	21848532	rs58570889	a	g	0.07	1.98	0.43	$4.89x10^{-6}$	+?	7.24	3.1	16.93	*MTAP*	intronic
chr9	111139461	rs71372609	ca	c	0.03	-2.64	0.57	$3.58x10^{-6}$	-?	0.07	0.02	0.22	*LOC105376219*	non-coding intronic
chr10	73604776	rs143580123	a	c	0.02	-2.88	0.63	$4.88x10^{-6}$	-?	0.06	0.02	0.19	*USP54*	intronic
chr11	99911908	rs17134658	t	g	0.04	-1.7	0.37	$4.51x10^{-6}$	--	0.18	0.09	0.38	*CNTN5*	intronic
chr11	28307404	chr11_28307404_C_T	t	c	NA	2.56	0.56	$4.67x10^{-6}$	+?	12.9	4.32	38.55	*METTL15*	NA
chr11	28212651	rs78280005	t	g	0.02	2.65	0.58	$4.77x10^{-6}$	+?	14.19	4.55	44.23	*METTL15*	intronic
chr11	7698038	rs142811167	t	c	0.01	4.47	0.98	$4.79x10^{-6}$	+?	87.06	12.84	590.36	*OVCH2*	intronic
chr14	92511954	rs140514603	a	g	0.02	-3.1	0.66	$2.90x10^{-6}$	-?	0.04	0.01	0.16	*RIN3*	5upstream
chr15	96309570	rs61037031	a	c	0.17	1.39	0.29	$1.74x10^{-6}$	+?	4.02	2.27	7.12	*NR2F2-AS1*	non-coding intronic
chr15	96309157	rs57751619	a	g	0.17	1.35	0.29	$2.49x10^{-6}$	+?	3.84	2.19	6.72	*NR2F2_AS1*	non-coding intronic
chr15	96299889	rs67631716	t	c	0.18	-1.35	0.29	$2.52x10^{-6}$	-?	0.26	0.15	0.46	*NR2F2_AS1*	non-coding intronic
chr15	96296956	rs4984430	t	c	0.18	1.35	0.29	$2.56x10^{-6}$	+?	3.86	2.2	6.77	*NR2F2_AS1*	non-coding intronic
chr15	96301872	rs4984534	c	g	0.18	-1.35	0.29	$2.56x10^{-6}$	-?	0.26	0.15	0.46	*NR2F2_AS1*	non-coding intronic
chr15	96319055	rs72751281	a	t	0.19	-1.44	0.31	$2.56x10^{-6}$	-?	0.24	0.13	0.43	*NR2F2_AS1*	non-coding intronic

Chr	Position	SNP ID	Allele 1	Allele 2	MAF	Effect	StdErr	P value	Direction	OR	lower_95CI	upper_95CI	Gene	Location
chr15	96297354	rs4984431	c	g	0.18	1.35	0.29	$2.57x10^{-6}$	+?	3.85	2.2	6.76	*NR2F2_AS1*	non-coding intronic
chr15	96302706	rs7166476	a	g	0.18	1.35	0.29	$2.59x10^{-6}$	+?	3.84	2.19	6.73	*NR2F2_AS1*	non-coding intronic
chr15	96291737	rs74699145	t	c	0.18	1.35	0.29	$2.69x10^{-6}$	+?	3.86	2.2	6.79	*NR2F2_AS1*	non-coding intronic
chr15	96301986	rs4984535	t	g	0.18	1.34	0.29	$2.75x10^{-6}$	+?	3.83	2.19	6.72	*NR2F2_AS1*	non-coding intronic
chr15	96287650	rs8030202	t	c	0.18	1.35	0.29	$2.91x10^{-6}$	+?	3.87	2.2	6.84	*NR2F2_AS1*	non-coding intronic
chr15	96290417	rs200005673	a	aaac	0.18	1.35	0.29	$2.94x10^{-6}$	+?	3.87	2.19	6.82	*NR2F2_AS1*	non-coding intronic
chr15	96320859	rs67688448	a	t	0.19	1.44	0.31	$3.08x10^{-6}$	+?	4.21	2.3	7.71	*NR2F2_AS1*	non-coding intronic
chr15	96320852	rs68071696	a	t	0.19	-1.44	0.31	$3.12x10^{-6}$	-?	0.24	0.13	0.43	*NR2F2_AS1*	non-coding intronic
chr15	96316173	rs55712637	a	g	0.19	-1.36	0.3	$4.94x10^{-6}$	-?	0.26	0.14	0.46	*NR2F2-AS1*	non-coding intronic
chr17	8041976	rs73972650	a	g	0.01	-2.09	0.41	$4.30x10^{-7}$	--	0.12	0.05	0.28	*ALOX15B*	intronic
chr17	8048560	rs7225107	a	g	0.01	-2.07	0.41	$5.10x10^{-7}$	--	0.13	0.06	0.28	*ALOX15B*	coding
chr17	8061912	rs8076459	t	g	0.01	2.09	0.42	$7.62x10^{-7}$	++	8.1	3.54	18.56	*NA*	NA
chr17	8050147	rs140958758	a	c	0.01	-3.41	0.74	$4.73x10^{-6}$	-?	0.03	0.01	0.14	*ALOX15B*	3downstream
chr18	5856605	rs56929051	c	g	0.05	2.72	0.58	$2.75x10^{-6}$	+?	15.21	4.87	47.46	NA	NA

Table S2 SNPs associated with VTE in the non-cancer population. Eighty five SNP associated with VTE in non cancer populations were identified from adult series and one pediatric study

**Supplementary Table S2. SNPs associated with VTE in otherwise healthy adult and paediatric patients**					
**SNP**	**Gene implicated**	**Risk allele**	***P* value**	**OR (95% CI)**	**Reference**
rs6025	*F5*	T	$3.6x10^{-137}$	2.93 (2.72-3.15)	(Germain *et al*; Heit *et al*; Hinds *et al*).
rs529565	*ABO*	C	$7.1x10^{-63}$	0.723 (0.70-0.75)	(Germain *et al*; Hinds *et al*)
rs4444878	*F11*	C	$7x10^{-28}$	0.81 (0.78-0.84)	(Hinds *et al*)
rs1799963	*F2*	A	$1.3x10^{-24}$	0.51 (0.46-0.58)	(Germain *et al*; Germain *et al*; Hinds *et al*)
rs7654093	*FGG*	T	$2x10^{-19}$	1.22 (1.17-1.27)	(Hinds *et al*)
rs114209171	*F8 (*upstream of *F8* gene, within *FUNDC2)*	T	a $7x10^{-13}$	1.15 (1.11-1.20)a	(Hinds *et al*)
rs9797861	*SLC44A2*	T	$6.1x10^{-9}$	1.15 (1.09-1.20)	(Hinds *et al*)
rs34234989	*PROCR*	D	$6.7x10^{-9}$	0.89 (0.85-0.92)	(Hinds *et al*)
rs72798544	*COX7A2L/KCNG3*	G	$1.9x10^{-7}$	0.73 (0.65-0.82)	(Hinds *et al*)
rs17490626	*TSPAN15*	G	$2.9x10^{-7}$	1.17 (1.10-1.24)	(Hinds *et al*)
rs113092656	*TMEM170B/ADTRP*	A	$4.4x10^{-7}$	0.73 (0.65-0.82)	(Hinds *et al*)
rs60942712	*EPHA3*	T	$8.0x10^{-7}$	1.21 (1.12-1.31)	(Hinds *et al*)
rs4524	*F5*	T	$2.65x10^{-11}$	1.2 (1.14-1.26)	(Germain *et al*)
rs2066865	*FGG*	A	$1.03x10^{-16}$	1.24 (1.18-1.31)	(Germain *et al*)
rs4253417	*F11*	C	$1.21x10^{-23}$	1.27 (1.22-1.34)	(Germain *et al*)
rs6087685	*PROCR*	C	$1.65x10^{-8}$	1.15 (1.10-1.21)	(Germain *et al*)
rs4602861	*ZFPM2*	A	$3.48x10^{-9}$ (discovery)	1.20 (1.13-1.27)	(Germain *et al*)

**Supplementary Table S2. SNPs associated with VTE in otherwise healthy adult and paediatric patients**					
**SNP**	**Gene implicated**	**Risk allele**	***P* value**	**OR (95% CI)**	**Reference**
rs78707713	*TSPAN15*	T	5.74×10^{-11} (discovery) subsequent validation 1.67×10^{-16}	1.42 (1.24-1.62, validation cohort)	(Germain *et al*)
rs2288904	*SLC44A2*	G	1.07×10^{-9} (discovery) [illegible]	1.28 (1.16-2.64, validation cohort)	(Germain *et al*)
rs16861990	*NME7*	C	4.9×10^{-9}	2.02 (1.66-2.45)	(Heit *et al*)
rs2519093	*ABO*	A	1.2×10^{-17}	1.69 (1.5-1.9)	(Heit *et al*)
rs495828	*ABO*	T	2.4×10^{-17}	1.65 (1.46-1.86)	(Heit *et al*)
rs8176719	*ABO*	G	5.7×10^{-16}	1.47 (1.32-1.64)	(Heit *et al*)
rs169713	*HIVEP1*	C	2.86×10^{-9}	1.2 (1.13-1.27)	(Morange *et al*)
rs867186	*PROCR*	G	$P<0.001$ c	1.22 (1.11-1.33)	(Dennis *et al*)
rs1120211	*C4BPA*	A	proxy SNPx10^{-10}	1.24 (1.03-1.53, validation cohort)	(Buil *et al*)
rs3813948	*C4BPB*	C	10^{-9}	see *SNP rs1120211*	(Buil *et al*)
rs2748331	*B3GAT2*	-	6.11×10^{-6}	not given	(Rühle *et al*)

Table S3. Lookup table of SNPs associated with VTE in non cancer patients in this meta-analysis. Published SNPs and those in complete linkage disequilibrium (r2=1) were looked up within the GWAS meta-analysis results from the ERASE and NOPHO cohorts. *P* value and odds ratio (OR) values are from the GWAS meta-analysis. The table is ordered according to chromosome and sequential position (based on human assembly GRCh37/hg19) within each chromosome. [a]The annotated gene was determined by cross-referencing Refseq, ensembl and UCSC database information (hg19,2015 update) accessed through SNPnexus (2012 update). The SNPnexus database (http://www.snp-nexus.org) is kept synchronised with the UCSC human genome annotation database (http://genome.ucsc.edu). Where there was discrepancy or the gene was uncertain, a search was performed manually using NCBI dbSNP build149. SNPs with a minor allele frequency (MAF) <1% were excluded. Functional annotation was determined using NCBI dbSNP build 149. SNPs with an odds ratio (OR) of 0.00 were excluded as were SNPs with an OR 95% confidence interval that included 1. *P* value thresholds are: <1 x10^{-5} is suggestive of an association and <5 x 10^{-8} is the threshold for genome-wide significance. CHR, chromosome.

Supplementary Table S3. Lookup table of SNPs associated with VTE in non cancer patients in this meta-analysis.

CHR	Position	Marker Name	Non effect allele	Effect allele	MAF	P-value	OR (95% CI)	Gene	Location
1	11856378	rs1801133	g	a	0.33	0.29	1.20 (0.85 - 1.69)	*MTHFR*	coding
1	67044724	rs6697088	g	c	0.42	0.08	0.60 (0.34 - 1.05)	*SGIP1*	intronic
1	67052164	rs1445575	c	t	0.43	0.06	1.70 (0.97 - 2.96)	*SGIP1*	intronic
1	67062885	rs964574	c	t	0.43	0.06	0.58 (0.33 - 1.02)	*SGIP1*	intronic
1	67065911	rs10889634	g	a	0.43	0.06	1.69 (0.97 - 2.93)	*SGIP1*	intronic
1	67066030	rs10789210	g	a	0.43	0.46	1.12 (0.82 - 1.53)	*SGIP1*	intronic
1	67066555	rs6588207	g	a	0.43	0.32	0.85 (0.62 - 1.16)	*SGIP1*	intronic
1	67068112	rs6689864	c	t	0.43	0.06	0.59 (0.33 - 1.02)	*SGIP1*	intronic
1	67068537	rs4655636	g	a	0.43	0.06	0.59 (0.34 - 1.02)	*SGIP1*	intronic
1	67070381	rs6696927	c	t	0.43	0.36	0.86 (0.63 - 1.18)	*SGIP1*	intronic
1	67072031	rs7524818	c	t	0.43	0.06	1.68 (0.97 - 2.92)	*SGIP1*	intronic
1	67073566	rs1562217	c	t	0.43	0.06	0.59 (0.34 - 1.03)	*SGIP1*	intronic
1	67083671	rs6656912	c	t	0.41	0.08	0.60 (0.34 - 1.05)	*SGIP1*	intronic
1	67086111	rs7532173	g	a	0.43	0.09	0.62 (0.36 - 1.07)	*SGIP1*	intronic
1	67106077	rs4655647	g	c	0.43	0.06	0.59 (0.34 - 1.03)	*SGIP1*	intronic

Supplementary Table S3. Lookup table of SNPs associated with VTE in non cancer patients in this meta-analysis.									
CHR	Position	Marker Name	Non effect allele	Effect allele	MAF	P-value	OR (95% CI)	Gene	Location
1	67114309	rs4655648	c	t	0.43	0.06	1.68 (0.97 - 2.93)	*SGIP1*	intronic
1	67116231	rs1329978	c	t	0.43	0.06	1.68 (0.97 - 2.93)	*SGIP1*	intronic
1	67121001	rs1536115	t	a	0.43	0.06	0.58 (0.33 - 1.02)	*SGIP1*	intronic
1	67121132	rs9662943	c	t	0.43	0.06	1.69 (0.97 - 2.94)	*SGIP1*	intronic
1	67121364	rs9662962	c	t	0.43	0.06	1.70 (0.98 - 2.96)	*SGIP1*	intronic
1	67121634	rs7521111	c	t	0.43	0.06	0.58 (0.33 - 1.01)	*SGIP1*	intronic
1	67121663	rs7521123	c	t	0.43	0.06	0.58 (0.33 - 1.01)	*SGIP1*	intronic
1	67122552	rs6656550	c	t	0.43	0.06	1.70 (0.98 - 2.96)	*SGIP1*	intronic
1	67122565	rs6680944	t	a	0.43	0.06	0.58 (0.33 - 1.01)	*SGIP1*	intronic
1	67123057	rs6681460	g	a	0.43	0.38	0.87 (0.63 - 1.18)	*SGIP1*	intronic
1	67123459	rs6659968	c	t	0.43	0.06	1.70 (0.98 - 2.96)	*SGIP1*	intronic
1	67123742	rs2031478	c	a	0.43	0.06	1.70 (0.98 - 2.96)	*SGIP1*	intronic
1	67126585	rs6670378	c	t	0.43	0.06	1.71 (0.98 - 2.99)	*SGIP1*	intronic
1	67127367	rs2872078	c	a	0.43	0.06	1.71 (0.98 - 2.99)	*SGIP1*	intronic
1	67129254	rs2147777	t	a	0.43	0.06	0.58 (0.33 - 1.01)	*SGIP1*	intronic
1	67129482	rs6680876	g	a	0.43	0.06	1.71 (0.98 - 2.99)	*SGIP1*	intronic
1	67129791	rs1325263	c	a	0.43	0.06	1.71 (0.98 - 2.99)	*SGIP1*	intronic
1	67138367	rs10889641	c	t	0.43	0.40	1.14 (0.83 - 1.55)	*SGIP1*	intronic
1	169099137	rs75112989	g	a	0.07	0.89	1.08 (0.36 - 3.22)	*ATP1B1*	intronic
1	169135127	rs16861990	c	a	0.07	0.91	0.93 (0.31 - 2.82)	*NME7*	intronic
1	169296864	rs1208327	c	t	0.07	0.76	0.90 (0.47 - 1.71)	*NME7*	intronic
1	169314430	rs1200063	c	t	0.07	0.87	1.09 (0.36 - 3.33)	*NME7*	intronic
1	169424098	rs1208135	g	a	0.07	0.88	1.09 (0.35 - 3.34)	*CCDC181*	intronic
1	169428944	rs1208134	c	t	0.07	0.67	0.86 (0.45 - 1.65)	*CCDC181*	intronic
1	169467654	rs1894692	g	a	0.02	0.20	0.39 (0.09 - 1.62)	-	-
1	169473503	rs10919178	c	t	0.27	0.54	0.89 (0.63 - 1.27)	-	-

Supplementary Table S3. Lookup table of SNPs associated with VTE in non cancer patients in this meta-analysis.									
CHR	Position	Marker Name	Non effect allele	Effect allele	MAF	P-value	OR (95% CI)	Gene	Location
1	169478654	rs974793	c	t	0.27	0.30	1.35 (0.76 - 2.37)	-	-
1	169480045	rs970741	c	t	0.27	0.30	0.74 (0.42 - 1.30)	*F5*	3downstream
1	169481950	rs2187952	g	a	0.27	0.55	1.11 (0.78 - 1.57)	*F5*	3utr
1	169483844	rs4656685	c	t	0.27	0.30	1.35 (0.76 - 2.37)	*F5*	intronic
1	169486501	rs9332667	g	c	0.27	0.30	1.35 (0.76 - 2.37)	*F5*	intronic
1	169489358	rs2227244	c	t	0.27	0.30	0.73 (0.42 - 1.30)	*F5*	intronic
1	169489512	rs2213866	g	a	0.27	0.29	0.73 (0.42 - 1.30)	*F5*	intronic
1	169489585	rs2213867	c	t	0.27	0.56	0.90 (0.63 - 1.27)	*F5*	intronic
1	169490592	rs9332655	g	a	0.27	0.55	0.89 (0.63 - 1.27)	*F5*	intronic
1	169492676	rs9332643	c	t	0.27	0.29	1.35 (0.76 - 2.38)	*F5*	intronic
1	169495433	rs9332635	c	t	0.27	0.29	0.73 (0.41 - 1.29)	*F5*	intronic
1	169497820	rs9332627	g	a	0.27	0.54	1.11 (0.78 - 1.58)	*F5*	intronic
1	169498181	rs2420373	c	t	0.27	0.29	1.35 (0.76 - 2.37)	*F5*	intronic
1	169499381	rs2187953	c	a	0.27	0.30	0.73 (0.42 - 1.30)	*F5*	intronic
1	169499951	rs9332620	c	t	0.27	0.30	1.35 (0.76 - 2.37)	*F5*	intronic
1	169500348	rs9332619	g	a	0.27	0.30	1.35 (0.76 - 2.37)	*F5*	intronic
1	169502533	rs6670393	c	a	0.27	0.29	0.73 (0.41 - 1.29)	*F5*	intronic
1	169503616	rs1121789	c	a	0.27	0.30	0.74 (0.42 - 1.30)	*F5*	intronic
1	169508739	rs3766109	t	a	0.27	0.31	0.74 (0.42 - 1.31)	*F5*	intronic
1	169511555	rs6032	c	t	0.27	0.31	0.74 (0.42 - 1.31)	*F5*	coding
1	169511734	rs4525	c	t	0.27	0.60	0.91 (0.64 - 1.29)	*F5*	coding
1	169511755	rs4524	c	t	0.27	0.60	0.91 (0.64 - 1.29)	*F5*	coding
1	169512027	rs6021	c	t	0.27	0.31	0.74 (0.42 - 1.31)	*F5*	coding
1	169512093	rs6017	g	a	0.27	0.60	0.91 (0.64 - 1.29)	*F5*	coding
1	169512120	rs6016	g	a	0.27	0.60	1.09 (0.77 - 1.56)	*F5*	coding
1	169512497	rs2239851	c	a	0.27	0.60	1.09 (0.77 - 1.55)	*F5*	intronic

Supplementary Table S3. Lookup table of SNPs associated with VTE in non cancer patients in this meta-analysis.									
**CHR**	**Position**	**Marker Name**	**Non effect allele**	**Effect allele**	**MAF**	**P-value**	**OR (95% CI)**	**Gene**	**Location**
1	169512562	rs6675244	c	t	0.27	0.31	0.74 (0.42 - 1.31)	*F5*	intronic
1	169512594	rs6662593	g	a	0.27	0.32	1.33 (0.75 - 2.35)	*F5*	intronic
1	169512651	rs6662696	g	a	0.27	0.31	1.34 (0.76 - 2.36)	*F5*	intronic
1	169512913	rs9332600	c	t	0.27	0.30	1.35 (0.76 - 2.38)	*F5*	intronic
1	169519049	rs6025	c	t	0.02	0.36	1.46 (0.64 - 3.31)	*F5*	coding
1	173562865	rs79491808	c	t	0.09	0.45	1.37 (0.60 - 3.12)	*SLC9C2*	intronic
1	173564989	rs74225380	c	a	0.09	0.45	0.72 (0.32 - 1.65)	*SLC9C2*	intronic
1	173565665	rs74225381	c	t	0.09	0.24	0.74 (0.44 - 1.22)	*SLC9C2*	intronic
1	173566402	rs74225382	g	a	0.09	0.45	0.72 (0.32 - 1.65)	*SLC9C2*	intronic
1	173591197	rs75584982	c	t	0.09	0.45	0.72 (0.32 - 1.65)	*ANKRD45*	intronic
1	173615965	rs7539322	c	t	0.09	0.24	1.34 (0.81 - 2.22)	*ANKRD45*	intronic
1	173616513	rs79714220	c	t	0.09	0.24	1.35 (0.82 - 2.22)	*ANKRD45*	intronic
1	173624194	rs115809785	c	t	0.09	0.45	1.37 (0.60 - 3.11)	*ANKRD45*	intronic
1	173625208	rs114290841	c	t	0.09	0.45	1.37 (0.60 - 3.11)	*ANKRD45*	intronic
1	173640519	rs74448174	c	t	0.09	0.45	0.72 (0.32 - 1.65)	*ANKRD45*	5upstream
1	173641886	rs58977252	g	a	0.09	0.45	1.37 (0.60 - 3.12)	*ANKRD45*	intronic
1	173651822	rs78002416	g	a	0.09	0.45	0.72 (0.31 - 1.66)	*ANKRD45*	intronic
1	173691842	rs77489136	g	t	0.08	0.44	0.70 (0.29 - 1.71)	*KLHL20*	intronic
1	173734769	rs3791022	g	a	0.09	0.33	0.78 (0.47 - 1.28)	*KLHL20*	intronic
1	173738295	rs61649485	c	a	0.09	0.46	0.73 (0.32 - 1.67)	*KLHL20*	intronic
1	173740290	rs78449808	g	c	0.09	0.46	0.73 (0.32 - 1.67)	*KLHL20*	intronic
1	173764414	rs74853670	g	a	0.09	0.46	0.73 (0.32 - 1.67)	-	-
1	173768826	rs16846418	g	c	0.09	0.46	0.73 (0.32 - 1.67)	*CENPL*	3utr
1	173796169	rs2068871	g	a	0.09	0.31	0.77 (0.47 - 1.27)	*DARS2*	intronic
1	173797752	rs75302147	g	a	0.09	0.46	0.73 (0.32 - 1.66)	*DARS2*	intronic
1	173811248	rs74202626	c	t	0.09	0.31	1.29 (0.78 - 2.11)	*DARS2*	intronic

Supplementary Table S3. Lookup table of SNPs associated with VTE in non cancer patients in this meta-analysis.									
**CHR**	**Position**	**Marker Name**	**Non effect allele**	**Effect allele**	**MAF**	**P-value**	**OR (95% CI)**	**Gene**	**Location**
1	173820593	rs16846433	g	a	0.09	0.31	1.29 (0.78 - 2.11)	*DARS2*	intronic
1	173830217	rs9970048	c	t	0.11	0.47	1.33 (0.61 - 2.91)	-	-
1	173831299	rs58994962	c	a	0.11	0.47	0.74 (0.34 - 1.63)	*GAS5*	3downstream
1	173831345	rs61262741	g	a	0.11	0.47	1.33 (0.61 - 2.91)	*GAS5*	3downstream
1	173832647	rs16846478	t	a	0.11	0.48	0.75 (0.34 - 1.64)	*GAS5*	3downstream
1	173834605	rs2235095	g	a	0.11	0.47	1.33 (0.61 - 2.91)	*GAS5*	non-coding intronic
1	173834632	rs6790	g	a	0.11	0.31	1.27 (0.79 - 2.06)	*GAS5*	non-coding
1	173837051	rs75315904	g	a	0.11	0.47	1.33 (0.61 - 2.91)	*ZBTB37*	5upstream
1	173845232	rs2065170	c	t	0.11	0.41	0.72 (0.33 - 1.56)	*ZBTB37*	intronic
1	173847020	rs57022387	g	t	0.11	0.31	1.28 (0.79 - 2.06)	*ZBTB37*	intronic
1	173856237	rs16846526	g	a	0.11	0.41	1.38 (0.63 - 3.00)	*ZBTB37*	3downstream
1	173860182	rs16846546	c	t	0.11	0.31	0.78 (0.48 - 1.26)	*ZBTB37*	3utr
1	173869111	rs16846561	g	c	0.11	0.39	0.71 (0.32 - 1.54)	-	-
1	173869569	rs2146372	g	c	0.11	0.39	1.40 (0.64 - 3.05)	-	-
1	173876705	rs2759328	c	t	0.11	0.39	1.40 (0.64 - 3.05)	*SERPINC1*	intronic
1	173881893	rs941988	c	t	0.11	0.38	1.41 (0.65 - 3.05)	*SERPINC1*	intronic
1	173885712	rs2227592	c	t	0.11	0.35	1.45 (0.66 - 3.16)	*SERPINC1*	intronic
1	173886216	rs2227589	c	t	0.11	0.27	1.31 (0.81 - 2.11)	*SERPINC1*	intronic
1	173891231	rs75713398	t	a	0.11	0.35	1.45 (0.66 - 3.16)	-	-
1	207269858	exm-rs3813948	c	t	0.08	0.95	0.97 (0.36 - 2.58)	-	-
2	42599605	rs72798544	g	t	0.03	0.45	0.59 (0.15 - 2.30)	-	-
2	151962991	rs4664951	c	a	0.48	0.71	1.06 (0.77 - 1.44)	-	-
2	151963175	rs4664952	c	t	0.48	0.85	1.05 (0.63 - 1.75)	-	-
2	151966336	rs1351930	t	a	0.47	0.90	1.03 (0.62 - 1.72)	-	-
2	151966685	rs10930435	g	a	0.47	0.90	0.96 (0.57 - 1.61)	-	-

Supplementary Table S3. Lookup table of SNPs associated with VTE in non cancer patients in this meta-analysis.									
**CHR**	**Position**	**Marker Name**	**Non effect allele**	**Effect allele**	**MAF**	**P-value**	**OR (95% CI)**	**Gene**	**Location**
2	151966879	rs11889216	c	a	0.47	0.90	0.96 (0.57 - 1.61)	-	-
2	151966912	rs11883594	c	t	0.47	0.90	1.03 (0.62 - 1.72)	-	-
2	151967300	rs1115140	c	t	0.47	0.90	0.96 (0.57 - 1.61)	-	-
2	151968356	rs2086914	g	a	0.47	0.90	1.03 (0.62 - 1.72)	-	-
2	151968650	rs4606923	c	a	0.47	0.90	0.96 (0.57 - 1.61)	-	-
2	151968704	rs3940993	c	a	0.47	0.90	0.96 (0.57 - 1.61)	-	-
2	151969632	rs10174574	c	t	0.47	0.90	1.03 (0.62 - 1.72)	-	-
2	151969979	rs1485117	c	t	0.47	0.90	0.96 (0.57 - 1.61)	-	-
2	161881944	rs113714975	g	a	0.3	0.73	1.10 (0.61 - 1.99)	-	-
2	161883435	rs34494741	c	a	0.3	0.72	0.89 (0.49 - 1.61)	-	-
2	161884425	rs4664393	t	a	0.3	0.73	0.90 (0.50 - 1.62)	-	-
2	161886103	rs2357982	g	c	0.3	0.75	1.09 (0.61 - 1.97)	-	-
2	161888412	rs2859653	g	c	0.3	0.73	0.90 (0.50 - 1.61)	-	-
2	161897159	rs197277	c	t	0.3	0.77	1.05 (0.75 - 1.46)	-	-
2	180216731	rs13403289	c	a	0.41	0.80	1.04 (0.74 - 1.46)	-	-
3	89047759	rs60942712	g	t	0.13	0.84	1.05 (0.65 - 1.69)	-	-
4	155501188	rs6825454	c	t	0.27	0.84	0.94 (0.52 - 1.69)	-	-
4	155503212	rs11099961	c	t	0.27	0.78	1.08 (0.60 - 1.95)	*FGA*	3downstream
4	155507590	rs6050	c	t	0.27	0.77	0.91 (0.51 - 1.64)	*FGA*	coding
4	155514879	rs13109457	g	a	0.26	0.39	1.15 (0.82 - 1.61)	-	-
4	155517842	rs35147053	g	a	0.26	0.85	0.94 (0.52 - 1.71)	-	-
4	155520930	rs7659024	g	a	0.25	0.66	1.07 (0.76 - 1.52)	-	-
4	155525276	rs2066865	g	a	0.25	0.66	1.07 (0.76 - 1.52)	*FGG*	3downstream
4	155525695	rs2066864	g	a	0.25	0.84	0.94 (0.51 - 1.72)	*FGG*	intronic
4	155527436	rs2066861	c	t	0.25	0.86	0.94 (0.51 - 1.74)	*FGG*	intronic
4	155535181	rs2066854	t	a	0.25	0.86	0.94 (0.51 - 1.74)	*FGG*	5upstream

Supplementary Table S3. Lookup table of SNPs associated with VTE in non cancer patients in this meta-analysis.									
**CHR**	**Position**	**Marker Name**	**Non effect allele**	**Effect allele**	**MAF**	**P-value**	**OR (95% CI)**	**Gene**	**Location**
4	155537321	rs12644950	g	a	0.25	0.54	1.11 (0.79 - 1.56)	-	-
4	155542248	rs7681423	c	t	0.26	0.82	0.93 (0.50 - 1.72)	-	-
4	155545072	rs7654093	t	a	0.25	0.91	1.03 (0.54 - 1.98)	-	-
4	187120211	rs13146272	c	a	0.36	0.45	0.88 (0.63 - 1.22)	*CYP4V2*	coding
4	187120697	rs2292425	g	a	0.36	0.99	1.00 (0.57 - 1.75)	*CYP4V2*	intronic
4	187187569	rs925451	g	a	0.37	0.20	1.23 (0.89 - 1.69)	*F11*	intronic
4	187199005	rs4253417	c	t	0.39	0.38	1.27 (0.74 - 2.18)	*F11*	intronic
4	187206249	rs3756011	c	a	0.39	0.33	1.16 (0.85 - 1.59)	*F11*	intronic
4	187207381	rs2289252	c	t	0.39	0.40	1.14 (0.83 - 1.56)	*F11*	intronic
5	51704512	rs1424905	c	t	0.38	0.96	1.01 (0.59 - 1.74)	-	-
5	51705503	rs62358259	g	a	0.37	0.70	0.89 (0.51 - 1.56)	-	-
5	51705534	rs12519037	c	t	0.37	0.71	0.89 (0.51 - 1.56)	-	-
5	51706906	rs6889746	g	a	0.38	0.87	0.97 (0.71 - 1.33)	-	-
5	51706979	rs6889897	g	a	0.38	0.69	1.12 (0.64 - 1.95)	-	-
5	51708894	rs34781462	g	a	0.37	0.73	0.90 (0.52 - 1.57)	-	-
5	51709218	rs6865723	c	t	0.38	0.63	0.92 (0.68 - 1.25)	-	-
5	51710521	rs34506741	c	a	0.37	0.73	0.90 (0.52 - 1.57)	-	-
6	6318795	rs5985	c	a	0.25	0.43	1.15 (0.81 - 1.63)	*F13A1*	coding
6	11615305	rs113092656	g	a	0.03	0.60	0.52 (0.04 - 5.91)	-	-
6	11919929	rs85219	g	a	0.21	0.39	0.72 (0.35 - 1.48)	-	-
6	11920517	rs169713	c	t	0.21	0.56	1.12 (0.76 - 1.65)	-	-
6	71623253	rs1304029	g	a	0.34	0.88	1.04 (0.59 - 1.82)	*B3GAT2*	intronic
6	71632028	rs2460696	c	t	0.33	0.85	0.94 (0.54 - 1.64)	*B3GAT2*	intronic
6	71721328	rs2748331	c	t	0.26	0.85	0.96 (0.67 - 1.37)	-	-
6	71820408	rs9455329	c	t	0.11	0.49	0.82 (0.47 - 1.42)	-	-
6	71823460	rs9455330	g	a	0.11	0.19	2.30 (0.65 - 8.08)	-	-

Supplementary Table S3. Lookup table of SNPs associated with VTE in non cancer patients in this meta-analysis.									
**CHR**	**Position**	**Marker Name**	**Non effect allele**	**Effect allele**	**MAF**	**P-value**	**OR (95% CI)**	**Gene**	**Location**
6	71826158	rs9446340	c	t	0.11	0.49	1.21 (0.70 - 2.10)	-	-
6	72666867	rs9293858	g	a	0.23	0.87	1.05 (0.56 - 1.97)	*RIMS1*	intronic
6	72667133	rs16881973	g	a	0.23	0.87	1.05 (0.56 - 1.97)	*RIMS1*	intronic
6	72669619	rs9446516	g	a	0.23	0.88	1.05 (0.56 - 1.96)	*RIMS1*	intronic
6	72682023	rs9446521	g	a	0.24	0.70	0.88 (0.47 - 1.65)	*RIMS1*	intronic
6	72983738	rs74543374	g	t	0.03	0.90	1.09 (0.25 - 4.71)	*RIMS1*	intronic
6	72984123	rs41265501	c	t	0.03	0.50	1.38 (0.53 - 3.58)	*RIMS1*	intronic
6	73015309	rs76132173	c	a	0.02	0.30	0.55 (0.18 - 1.67)	*RIMS1*	intronic
6	80156259	rs12204683	c	t	0.29	0.83	0.96 (0.68 - 1.35)	*LOC100506851*	intronic
6	80157561	rs2629475	c	a	0.29	0.71	1.11 (0.62 - 2.00)	*LOC100506851*	intronic
7	32189540	rs11975235	c	a	0.44	NA		*PDE1C*	intronic
8	106590706	rs4602861	g	a	0.25	0.45	0.78 (0.41 - 1.47)	*ZFPM2*	intronic
9	136137065	rs687621	g	a	0.32	0.61	0.87 (0.52 - 1.46)	*ABO*	intronic
9	136137106	rs687289	g	a	0.32	0.65	1.07 (0.78 - 1.47)	*ABO*	intronic
9	136139265	rs657152	c	a	0.33	0.37	1.15 (0.84 - 1.57)	*ABO*	intronic
9	136141870	rs2519093	c	t	0.17	0.29	1.37 (0.75 - 2.51)	*ABO*	intronic
9	136142203	rs514659	c	a	0.32	0.44	0.81 (0.49 - 1.36)	*ABO*	intronic
9	136142355	rs643434	g	a	0.33	0.29	1.31 (0.79 - 2.18)	*ABO*	intronic
9	136143212	rs544873	g	a	0.33	0.29	1.31 (0.78 - 2.18)	*ABO*	intronic
9	136143372	rs545971	c	t	0.32	0.44	1.22 (0.73 - 2.03)	*ABO*	intronic
9	136143442	rs612169	g	a	0.32	0.44	0.81 (0.49 - 1.36)	*ABO*	intronic
9	136144873	rs491626	c	t	0.32	0.44	1.22 (0.73 - 2.03)	*ABO*	intronic
9	136144960	rs492488	g	a	0.32	0.44	1.22 (0.73 - 2.03)	*ABO*	intronic
9	136144994	rs493246	g	a	0.32	0.44	1.22 (0.73 - 2.03)	*ABO*	intronic
9	136145240	rs495203	c	t	0.32	0.44	1.22 (0.73 - 2.03)	*ABO*	intronic
9	136145974	rs2769071	g	a	0.32	0.44	0.81 (0.49 - 1.36)	*ABO*	intronic

Supplementary Table S3. Lookup table of SNPs associated with VTE in non cancer patients in this meta-analysis.									
**CHR**	**Position**	**Marker Name**	**Non effect allele**	**Effect allele**	**MAF**	**P-value**	**OR (95% CI)**	**Gene**	**Location**
9	136146077	rs676996	g	t	0.32	0.44	0.81 (0.49 - 1.36)	*ABO*	intronic
9	136146227	rs676457	t	a	0.32	0.44	0.81 (0.49 - 1.36)	*ABO*	intronic
9	136146664	rs674302	t	a	0.32	0.44	1.22 (0.73 - 2.03)	*ABO*	intronic
9	136147160	rs554833	c	t	0.32	0.45	1.21 (0.73 - 2.02)	*ABO*	intronic
9	136149500	rs529565	c	t	0.32	0.55	0.90 (0.66 - 1.24)	*ABO*	intronic
9	136149830	rs532436	g	a	0.17	0.51	1.13 (0.78 - 1.64)	*ABO*	intronic
9	136151806	rs600038	c	t	0.19	0.17	0.66 (0.37 - 1.18)	*ABO*	5upstream
9	136154168	rs579459	c	t	0.19	0.35	0.84 (0.58 - 1.20)	-	-
9	136154304	rs649129	c	t	0.19	0.16	1.51 (0.84 - 2.71)	-	-
9	136154867	rs495828	g	t	0.19	0.24	1.39 (0.80 - 2.43)	-	-
9	136155000	rs635634	c	t	0.17	0.24	1.42 (0.78 - 2.59)	-	-
10	71218646	rs17490626	g	c	0.12	0.96	0.98 (0.42 - 2.24)	*TSPAN15*	intronic
10	71245276	rs78707713	c	t	0.12	0.98	0.98 (0.43 - 2.24)	*TSPAN15*	intronic
13	40741907	rs9315762	c	t	0.19	0.44	1.29 (0.67 - 2.46)	-	-
13	109380726	rs10492418	c	a	0.45	0.81	1.06 (0.63 - 1.79)	*MYO16*	intronic
16	80187374	rs10514487	c	t	0.05	0.39	1.34 (0.68 - 2.64)	*LOC105371357*	intronic
16	80191464	rs12102687	g	a	0.05	0.65	1.38 (0.33 - 5.62)	*LOC102724084*	intronic
16	80191694	rs12103210	g	a	0.05	0.61	0.69 (0.16 - 2.87)	*LOC102724084*	intronic
16	80192405	rs8045802	t	a	0.05	0.66	0.72 (0.17 - 2.95)	*LOC102724084*	intronic
16	80192605	rs8060581	g	c	0.05	0.66	0.72 (0.17 - 2.95)	*LOC102724084*	intronic
16	80192921	rs12598278	c	a	0.05	0.42	0.75 (0.38 - 1.49)	*LOC102724084*	intronic
16	80198653	rs6564743	g	a	0.05	0.68	1.34 (0.33 - 5.41)	*LOC102724084*	intronic
19	10738639	rs3087969	c	t	0.23	0.45	0.85 (0.55 - 1.29)	*SLC44A2*	coding
19	10738836	rs8109681	g	a	0.23	0.64	0.85 (0.43 - 1.66)	*SLC44A2*	intronic
19	10739073	rs8108741	g	a	0.23	0.64	1.17 (0.60 - 2.29)	*SLC44A2*	intronic
19	10739143	rs8110055	c	a	0.23	0.63	0.84 (0.43 - 1.65)	*SLC44A2*	intronic

Supplementary Table S3. Lookup table of SNPs associated with VTE in non cancer patients in this meta-analysis.

CHR	Position	Marker Name	Non effect allele	Effect allele	MAF	P-value	OR (95% CI)	Gene	Location
19	10740871	rs4548995	g	c	0.23	0.63	1.17 (0.60 - 2.30)	*SLC44A2*	intronic
19	10742170	rs2288904	g	a	0.23	0.64	0.85 (0.43 - 1.65)	*SLC44A2*	coding
19	10742287	rs1560711	c	t	0.23	0.64	1.17 (0.60 - 2.28)	*SLC44A2*	intronic
19	55535069	rs1671193	g	a	0.19	0.89	0.95 (0.49 - 1.85)	*GP6*	intronic
19	55535112	rs1654418	c	t	0.19	0.89	0.95 (0.49 - 1.84)	*GP6*	intronic
19	55535289	rs11671922	g	a	0.19	0.61	0.90 (0.60 - 1.33)	*GP6*	intronic
19	55535418	rs11668161	g	t	0.19	0.89	0.95 (0.49 - 1.84)	*GP6*	intronic
19	55535482	rs11668169	c	t	0.19	0.88	0.95 (0.49 - 1.84)	*GP6*	intronic
19	55535881	rs1654419	g	a	0.19	0.89	0.95 (0.49 - 1.84)	*GP6*	intronic
19	55536206	rs1654420	t	a	0.19	0.89	1.04 (0.54 - 2.03)	*GP6*	intronic
19	55536595	rs1613662	g	a	0.16	0.59	1.12 (0.73 - 1.71)	*GP6*	coding
19	55537942	rs2886412	c	t	0.19	0.89	0.95 (0.49 - 1.84)	*GP6*	intronic
19	55538980	rs1654425	c	t	0.16	0.81	0.91 (0.42 - 1.96)	*GP6*	coding
19	55539548	rs1671196	c	t	0.19	0.89	1.04 (0.53 - 2.03)	*GP6*	intronic
19	55539834	rs1671198	c	t	0.19	0.90	1.04 (0.53 - 2.03)	*GP6*	intronic
19	55539868	rs1671199	g	a	0.19	0.91	0.96 (0.49 - 1.86)	*GP6*	intronic
20	15997027	rs1237878	g	a	0.31	0.76	1.09 (0.63 - 1.87)	*MACROD2*	intronic
20	16003406	rs6080100	c	t	0.31	0.73	0.90 (0.52 - 1.56)	*MACROD2*	intronic
20	17545376	rs74181970	g	a	0.09	0.72	1.15 (0.51 - 2.59)	*BFSP1*	intronic
20	17549620	rs35384212	g	t	0.09	0.99	1.00 (0.41 - 2.46)	*DSTN*	5upstream
20	17550020	rs74181972	g	t	0.09	0.72	0.86 (0.38 - 1.92)	*DSTN*	5upstream
20	17559663	rs13041238	g	a	0.09	0.83	1.06 (0.61 - 1.81)	*DSTN*	intronic
20	17563138	rs7271384	g	c	0.09	0.71	1.16 (0.52 - 2.60)	*DSTN*	intronic
20	17563241	rs7264490	c	t	0.09	0.71	0.85 (0.38 - 1.91)	*DSTN*	intronic
20	17566069	rs17791782	t	a	0.08	1.00	0.99 (0.41 - 2.43)	*DSTN*	intronic
20	17577040	rs13044119	t	a	0.09	0.72	0.86 (0.38 - 1.92)	*DSTN*	intronic

Supplementary Table S3. Lookup table of SNPs associated with VTE in non cancer patients in this meta-analysis.									
**CHR**	**Position**	**Marker Name**	**Non effect allele**	**Effect allele**	**MAF**	**P-value**	**OR (95% CI)**	**Gene**	**Location**
20	17581192	rs13038541	c	t	0.09	0.83	1.06 (0.62 - 1.81)	*DSTN*	intronic
20	33544277	rs34174778	g	c	0.08	0.85	1.09 (0.41 - 2.90)	*GSS*	5upstream
20	33544973	rs35552264	t	a	0.08	0.83	1.11 (0.41 - 2.96)	*GSS*	5upstream
20	33545616	rs17310467	g	a	0.08	0.71	1.10 (0.65 - 1.85)	*GSS*	5upstream
20	33549407	rs55909363	c	t	0.08	0.83	0.89 (0.33 - 2.38)	*MYH7B*	intronic
20	33549887	rs55696836	g	a	0.08	0.86	0.91 (0.34 - 2.41)	*MYH7B*	intronic
20	33551462	rs80170004	g	a	0.08	0.87	0.92 (0.34 - 2.42)	*MYH7B*	intronic
20	33552642	rs17401737	c	a	0.08	0.84	0.90 (0.34 - 2.40)	*MYH7B*	intronic
20	33555815	rs75635914	c	t	0.08	0.86	0.91 (0.34 - 2.42)	*MYH7B*	intronic
20	33558839	rs75866240	c	t	0.08	0.86	0.91 (0.34 - 2.42)	*MYH7B*	intronic
20	33560314	rs73905019	c	a	0.08	0.90	0.94 (0.36 - 2.46)	*MYH7B*	intronic
20	33561495	rs74599371	g	a	0.08	0.90	0.93 (0.35 - 2.45)	*MYH7B*	intronic
20	33562476	rs79197732	c	t	0.08	0.90	0.93 (0.35 - 2.46)	*MYH7B*	intronic
20	33563911	rs76191812	g	a	0.08	0.87	0.92 (0.34 - 2.43)	*MYH7B*	intronic
20	33564738	rs77437249	g	a	0.08	0.86	0.91 (0.34 - 2.42)	*MYH7B*	intronic
20	33565528	rs117616040	g	c	0.08	0.91	0.94 (0.35 - 2.49)	*MYH7B*	intronic
20	33570007	rs7269138	c	t	0.08	0.85	1.09 (0.41 - 2.89)	*MYH7B*	intronic
20	33574458	rs8118978	c	a	0.08	0.90	1.06 (0.40 - 2.78)	*MYH7B*	intronic
20	33576205	rs7261167	g	a	0.08	0.88	0.92 (0.35 - 2.42)	*MYH7B*	intronic
20	33585437	rs55734215	c	t	0.08	0.90	0.94 (0.35 - 2.46)	*MYH7B*	coding
20	33587596	rs80109502	g	a	0.08	0.90	0.93 (0.35 - 2.46)	*MYH7B*	coding
20	33591627	rs2295700	g	a	0.08	0.86	0.91 (0.34 - 2.42)	*TRPC4AP*	intronic
20	33592148	rs73905041	g	a	0.08	0.92	0.95 (0.36 - 2.47)	*TRPC4AP*	intronic
20	33592588	rs75537616	c	t	0.08	0.90	0.93 (0.35 - 2.46)	*TRPC4AP*	intronic
20	33594959	rs17317888	g	t	0.08	0.90	0.93 (0.35 - 2.46)	*TRPC4AP*	intronic
20	33595913	rs17092215	c	t	0.08	0.92	0.95 (0.36 - 2.47)	*TRPC4AP*	intronic

Supplementary Table S3. Lookup table of SNPs associated with VTE in non cancer patients in this meta-analysis.									
**CHR**	**Position**	**Marker Name**	**Non effect allele**	**Effect allele**	**MAF**	**P-value**	**OR (95% CI)**	**Gene**	**Location**
20	33598612	rs6579208	c	t	0.08	0.92	0.95 (0.36 - 2.47)	*TRPC4AP*	intronic
20	33598789	rs8123978	c	t	0.08	0.90	0.93 (0.35 - 2.46)	*TRPC4AP*	intronic
20	33599403	rs7273734	g	c	0.08	0.92	0.95 (0.36 - 2.47)	*TRPC4AP*	intronic
20	33601498	rs75535620	c	t	0.08	0.90	1.06 (0.40 - 2.79)	*TRPC4AP*	intronic
20	33605098	rs10485508	c	t	0.08	0.90	0.93 (0.35 - 2.46)	*TRPC4AP*	intronic
20	33605802	rs6579210	c	t	0.08	0.92	0.95 (0.36 - 2.47)	*TRPC4AP*	intronic
20	33605857	rs6579211	g	a	0.08	0.59	0.86 (0.51 - 1.45)	*TRPC4AP*	intronic
20	33608616	rs7274866	g	a	0.08	0.92	1.05 (0.40 - 2.73)	*TRPC4AP*	intronic
20	33610992	rs7271729	c	t	0.08	0.89	0.93 (0.35 - 2.45)	*TRPC4AP*	intronic
20	33618606	rs78055011	g	a	0.08	0.90	0.93 (0.35 - 2.46)	*TRPC4AP*	intronic
20	33623522	rs76507298	t	a	0.08	0.90	1.06 (0.40 - 2.79)	*TRPC4AP*	intronic
20	33626005	rs56363533	g	c	0.08	0.92	0.95 (0.36 - 2.47)	*TRPC4AP*	intronic
20	33629610	rs55946144	c	t	0.08	0.92	1.05 (0.40 - 2.73)	*TRPC4AP*	intronic
20	33636219	rs8118005	g	c	0.08	0.92	0.95 (0.36 - 2.47)	*TRPC4AP*	intronic
20	33639256	rs8124662	g	a	0.08	0.92	0.95 (0.36 - 2.47)	*TRPC4AP*	intronic
20	33641220	rs11907438	t	a	0.08	0.92	0.95 (0.36 - 2.47)	*TRPC4AP*	intronic
20	33642480	rs8117847	c	t	0.08	0.59	0.86 (0.51 - 1.45)	*TRPC4AP*	intronic
20	33645709	rs9941751	g	a	0.08	0.92	1.05 (0.40 - 2.73)	*TRPC4AP*	intronic
20	33646841	rs2889873	g	a	0.08	0.86	0.91 (0.34 - 2.42)	*TRPC4AP*	intronic
20	33647503	rs114007205	g	t	0.08	0.89	0.93 (0.35 - 2.45)	*TRPC4AP*	intronic
20	33649376	rs7263253	c	t	0.08	0.90	1.06 (0.40 - 2.79)	*TRPC4AP*	intronic
20	33649593	rs7268447	g	c	0.08	0.89	0.93 (0.35 - 2.45)	*TRPC4AP*	intronic
20	33651453	rs75165171	g	a	0.08	0.90	0.93 (0.35 - 2.46)	*TRPC4AP*	intronic
20	33651621	rs117573845	g	a	0.08	0.90	1.06 (0.40 - 2.79)	*TRPC4AP*	intronic
20	33652964	rs717593	g	c	0.08	0.90	0.93 (0.35 - 2.46)	*TRPC4AP*	intronic
20	33654584	rs11167254	c	t	0.08	0.92	0.95 (0.36 - 2.47)	*TRPC4AP*	intronic

Supplementary Table S3. Lookup table of SNPs associated with VTE in non cancer patients in this meta-analysis.									
**CHR**	**Position**	**Marker Name**	**Non effect allele**	**Effect allele**	**MAF**	**P-value**	**OR (95% CI)**	**Gene**	**Location**
20	33654689	rs79439610	c	a	0.08	0.90	0.93 (0.35 - 2.46)	*TRPC4AP*	intronic
20	33655535	rs76363448	c	t	0.08	0.90	1.06 (0.40 - 2.79)	*TRPC4AP*	intronic
20	33658658	rs74543591	c	t	0.08	0.90	1.06 (0.40 - 2.79)	*TRPC4AP*	intronic
20	33667424	rs78202808	c	t	0.08	0.92	1.04 (0.40 - 2.72)	*TRPC4AP*	intronic
20	33667783	rs117236853	c	a	0.08	0.90	0.93 (0.35 - 2.46)	*TRPC4AP*	intronic
20	33671725	rs76850134	c	a	0.08	0.90	1.06 (0.40 - 2.79)	*TRPC4AP*	intronic
20	33673843	rs11907594	c	t	0.08	0.92	0.95 (0.36 - 2.47)	*TRPC4AP*	intronic
20	33676109	rs8121957	c	t	0.08	0.92	1.04 (0.40 - 2.72)	*TRPC4AP*	intronic
20	33677164	rs11905354	g	a	0.08	0.92	0.95 (0.36 - 2.47)	*TRPC4AP*	intronic
20	33678732	rs73903009	g	a	0.08	0.92	0.95 (0.36 - 2.47)	*TRPC4AP*	intronic
20	33682570	rs6060222	c	a	0.09	0.95	1.02 (0.40 - 2.60)	*TRPC4AP*	5upstream
20	33684909	rs7261312	t	a	0.09	0.95	1.02 (0.40 - 2.61)	-	-
20	33689308	rs6060230	c	t	0.09	0.60	0.86 (0.51 - 1.45)	-	-
20	33689941	rs76648234	t	a	0.08	0.91	0.94 (0.36 - 2.46)	-	-
20	33691652	rs6060235	g	a	0.09	0.90	1.06 (0.41 - 2.73)	-	-
20	33692261	rs8117100	c	t	0.08	0.89	1.06 (0.40 - 2.79)	-	-
20	33692618	rs6060236	c	a	0.09	0.56	0.85 (0.51 - 1.43)	-	-
20	33693175	rs117316403	g	t	0.09	0.88	1.07 (0.41 - 2.82)	-	-
20	33693177	rs112777695	g	t	0.09	0.88	1.07 (0.41 - 2.80)	-	-
20	33693582	rs7270326	c	t	0.09	0.96	1.02 (0.40 - 2.60)	-	-
20	33693650	rs17319967	g	c	0.09	0.91	0.94 (0.36 - 2.43)	-	-
20	33699435	rs6060244	g	a	0.09	0.86	0.91 (0.35 - 2.38)	-	-
20	33699625	rs6060245	g	a	0.09	0.86	1.09 (0.41 - 2.84)	-	-
20	33700717	rs28469723	g	a	0.09	0.86	1.09 (0.41 - 2.84)	-	-
20	33701107	rs6060246	g	a	0.09	0.86	1.09 (0.41 - 2.84)	*EDEM2*	3downstream
20	33702104	rs79341738	c	t	0.09	0.86	1.09 (0.41 - 2.84)	*EDEM2*	3downstream

Supplementary Table S3. Lookup table of SNPs associated with VTE in non cancer patients in this meta-analysis.									
**CHR**	**Position**	**Marker Name**	**Non effect allele**	**Effect allele**	**MAF**	**P-value**	**OR (95% CI)**	**Gene**	**Location**
20	33702280	rs79438986	g	t	0.08	0.88	1.07 (0.41 - 2.80)	*EDEM2*	3downstream
20	33702998	rs75888794	g	c	0.09	0.85	0.91 (0.35 - 2.38)	*EDEM2*	3downstream
20	33703134	rs17092297	g	a	0.09	0.59	0.86 (0.51 - 1.44)	*EDEM2*	3downstream
20	33707177	rs111641740	c	t	0.09	0.86	0.91 (0.34 - 2.40)	*EDEM2*	intronic
20	33720920	rs11908683	c	t	0.08	0.42	1.24 (0.73 - 2.11)	*EDEM2*	intronic
20	33721261	rs75287019	g	c	0.08	0.88	1.07 (0.41 - 2.81)	*EDEM2*	intronic
20	33722863	rs2295888	g	a	0.08	0.43	1.23 (0.73 - 2.08)	*EDEM2*	intronic
20	33724221	rs75648520	c	t	0.08	0.87	0.92 (0.35 - 2.42)	*EDEM2*	intronic
20	33724758	rs73903017	g	a	0.08	0.91	1.05 (0.40 - 2.73)	*EDEM2*	intronic
20	33726150	rs60866116	c	t	0.08	0.91	1.05 (0.40 - 2.73)	*EDEM2*	intronic
20	33726873	rs8119827	c	t	0.08	0.89	0.93 (0.35 - 2.43)	*EDEM2*	intronic
20	33727779	rs73903019	g	t	0.08	0.92	0.94 (0.36 - 2.46)	*EDEM2*	intronic
20	33729442	rs11906318	c	a	0.08	0.50	1.20 (0.70 - 2.03)	*EDEM2*	intronic
20	33729477	rs11908232	g	c	0.08	0.92	1.04 (0.40 - 2.71)	*EDEM2*	intronic
20	33729479	rs11908100	g	a	0.08	0.44	0.81 (0.47 - 1.37)	*EDEM2*	intronic
20	33731010	rs12105996	g	a	0.08	0.93	0.95 (0.36 - 2.47)	*EDEM2*	intronic
20	33731437	rs57690120	t	a	0.08	0.93	1.04 (0.40 - 2.71)	*EDEM2*	intronic
20	33731484	rs79048371	t	a	0.08	0.88	0.92 (0.35 - 2.44)	*EDEM2*	intronic
20	33733641	rs114948279	g	a	0.08	0.89	0.93 (0.35 - 2.45)	*EDEM2*	intronic
20	33737661	rs11907010	c	t	0.08	0.43	0.80 (0.47 - 1.37)	-	-
20	33739456	rs80277764	c	t	0.08	0.89	0.93 (0.35 - 2.45)	-	-
20	33746668	rs74626382	g	t	0.08	0.89	0.93 (0.35 - 2.45)	-	-
20	33759272	rs2069940	g	c	0.08	0.85	1.09 (0.40 - 2.93)	*PROCR*	5upstream
20	33764554	rs867186	g	a	0.08	0.40	1.25 (0.74 - 2.14)	*PROCR*	coding
20	33767770	rs11907011	c	t	0.08	0.40	0.79 (0.46 - 1.35)	*PROCR*	intronic
20	33768523	rs7265317	c	t	0.08	0.86	1.09 (0.40 - 2.92)	*PROCR*	intronic

Supplementary Table S3. Lookup table of SNPs associated with VTE in non cancer patients in this meta-analysis.									
CHR	Position	Marker Name	Non effect allele	Effect allele	MAF	P-value	OR (95% CI)	Gene	Location
20	33769926	rs945961	g	a	0.08	0.86	1.09 (0.40 - 2.92)	*PROCR*	intronic
20	33773375	rs7263203	c	a	0.08	0.86	1.09 (0.40 - 2.92)	*PROCR*	intronic
20	33773630	rs117249133	g	a	0.08	0.81	1.13 (0.41 - 3.13)	*PROCR*	intronic
20	33775200	rs11167260	g	a	0.09	0.39	0.79 (0.46 - 1.34)	*PROCR*	intronic
20	33775369	rs10747514	g	a	0.28	0.81	0.95 (0.67 - 1.35)	*PROCR*	intronic
20	33777612	rs6087685	g	c	0.28	0.64	0.86 (0.46 - 1.60)	*PROCR*	intronic
20	33783053	rs78967641	g	t	0.08	0.86	0.91 (0.34 - 2.45)	*PROCR*	intronic
20	33784021	rs11906148	c	a	0.08	0.88	0.92 (0.34 - 2.49)	*PROCR*	intronic
20	33786677	rs117802529	c	t	0.08	0.86	0.91 (0.34 - 2.45)	*PROCR*	intronic
20	33793159	rs6088764	c	a	0.28	0.88	0.97 (0.68 - 1.37)	*PROCR*	intronic
20	33794378	rs6060308	g	a	0.27	0.79	0.91 (0.49 - 1.71)	*PROCR*	intronic
22	18437382	rs12158906	g	a	0.25	0.14	0.65 (0.37 - 1.14)	*MICAL3*	intronic
22	18438314	rs2099942	c	a	0.25	0.14	0.65 (0.37 - 1.14)	*MICAL3*	intronic
22	18438559	rs2099943	g	a	0.25	0.18	0.78 (0.55 - 1.12)	*MICAL3*	intronic
22	18438578	rs2099944	g	a	0.25	0.13	1.53 (0.87 - 2.70)	*MICAL3*	intronic
22	18438642	rs2099945	g	c	0.25	0.13	1.54 (0.87 - 2.70)	*MICAL3*	intronic
22	18439598	rs1076539	g	a	0.25	0.13	0.64 (0.36 - 1.13)	*MICAL3*	intronic
22	18439958	rs1076540	c	t	0.25	0.13	1.54 (0.87 - 2.71)	*MICAL3*	intronic
22	18440026	rs1076541	c	t	0.25	0.11	1.57 (0.89 - 2.76)	*MICAL3*	intronic
22	18443287	rs11705221	c	t	0.25	0.13	0.64 (0.36 - 1.13)	*MICAL3*	intronic
22	18443404	rs13053667	g	a	0.25	0.17	1.28 (0.89 - 1.82)	*MICAL3*	intronic
22	18443699	rs5992917	g	t	0.25	0.18	1.27 (0.89 - 1.82)	*MICAL3*	intronic
22	18443818	rs5992918	g	c	0.25	0.13	0.64 (0.36 - 1.13)	*MICAL3*	intronic
22	18443855	rs5992919	g	a	0.25	0.16	0.77 (0.54 - 1.10)	*MICAL3*	intronic
22	18444657	rs7286607	c	t	0.25	0.18	0.78 (0.55 - 1.12)	*MICAL3*	intronic
22	18444675	rs1110659	c	t	0.25	0.13	0.64 (0.36 - 1.13)	*MICAL3*	intronic

Supplementary Table S3. Lookup table of SNPs associated with VTE in non cancer patients in this meta-analysis.

CHR	Position	Marker Name	Non effect allele	Effect allele	MAF	P-value	OR (95% CI)	Gene	Location
22	18444693	rs1110660	c	a	0.25	0.13	0.64 (0.36 - 1.13)	*MICAL3*	intronic
22	18444908	rs1110662	g	a	0.25	0.13	1.55 (0.88 - 2.72)	*MICAL3*	intronic
22	18445263	rs5992921	c	t	0.25	0.18	1.27 (0.89 - 1.82)	*MICAL3*	intronic
22	18445287	rs5992922	g	a	0.25	0.12	0.63 (0.36 - 1.12)	*MICAL3*	intronic
22	18445288	rs7285566	g	a	0.24	0.11	1.59 (0.89 - 2.80)	*MICAL3*	intronic
22	18445709	rs7286219	g	c	0.25	0.13	1.55 (0.88 - 2.72)	*MICAL3*	intronic
22	18446024	rs13057203	g	a	0.25	0.13	0.64 (0.36 - 1.13)	*MICAL3*	intronic
22	18446212	rs11704733	g	t	0.25	0.13	0.64 (0.36 - 1.13)	*MICAL3*	intronic
22	18446318	rs1079576	c	a	0.25	0.13	1.55 (0.88 - 2.72)	*MICAL3*	intronic
22	18446452	rs1076542	g	a	0.25	0.13	1.55 (0.88 - 2.72)	*MICAL3*	intronic
22	18446490	rs1076543	c	t	0.25	0.13	0.64 (0.36 - 1.13)	*MICAL3*	intronic
22	18446565	rs1076544	g	a	0.25	0.13	1.55 (0.88 - 2.72)	*MICAL3*	intronic
22	18447114	rs5992135	c	t	0.25	0.13	1.55 (0.88 - 2.72)	*MICAL3*	intronic
22	18448113	rs1978968	c	t	0.25	0.12	1.55 (0.88 - 2.73)	*MICAL3*	intronic
22	18449038	rs7291169	g	a	0.25	0.13	0.64 (0.36 - 1.13)	*MICAL3*	intronic
22	18449543	rs11704059	g	a	0.25	0.13	0.64 (0.36 - 1.13)	*MICAL3*	intronic
22	18449876	rs5992926	g	a	0.25	0.13	0.64 (0.36 - 1.13)	*MICAL3*	intronic
22	18449927	rs5013014	c	t	0.25	0.13	0.64 (0.36 - 1.13)	*MICAL3*	intronic
22	18450116	rs5013013	c	t	0.25	0.13	0.64 (0.36 - 1.13)	*MICAL3*	intronic
22	18450149	rs5013012	g	a	0.25	0.13	0.64 (0.36 - 1.13)	*MICAL3*	intronic
22	18450287	rs11704827	t	a	0.25	0.13	0.64 (0.36 - 1.13)	*MICAL3*	intronic
22	18451391	rs4410378	g	a	0.25	0.13	1.55 (0.88 - 2.72)	*MICAL3*	intronic
22	18451438	rs5992928	c	a	0.25	0.13	0.64 (0.36 - 1.14)	*MICAL3*	intronic
22	18451554	rs28434757	g	a	0.25	0.14	1.53 (0.87 - 2.70)	*MICAL3*	Intronic

Buil A, Trégouët D-A, Souto JC, Saut N, Germain M, Rotival M, Tiret L, Cambien F, Lathrop M, Zeller T (2010) C4BPB/C4BPA is a new susceptibility locus for venous thrombosis with unknown protein S–independent mechanism: results from genome-wide association and gene expression analyses followed by case-control studies. *Blood* **115**(23)**:** 4644-4650

Dennis J, Johnson CY, Adediran AS, De Andrade M, Heit JA, Morange P-E, Trégouët D-A, Gagnon F (2012) The endothelial protein C receptor (PROCR) Ser219Gly variant and risk of common thrombotic disorders: a HuGE review and meta-analysis of evidence from observational studies. *Blood* **119**(10)**:** 2392-2400

Germain M, Chasman DI, De Haan H, Tang W, Lindström S, Weng L-C, De Andrade M, De Visser MC, Wiggins KL, Suchon P (2015) Meta-analysis of 65,734 individuals identifies TSPAN15 and SLC44A2 as two susceptibility loci for venous thromboembolism. *The American Journal of Human Genetics* **96**(4)**:** 532-542

Germain M, Saut N, Oudot-Mellakh T, Letenneur L, Dupuy A-M, Bertrand M, Alessi M-C, Lambert J-C, Zelenika D, Emmerich J (2012) Caution in interpreting results from imputation analysis when linkage disequilibrium extends over a large distance: a case study on venous thrombosis. *PloS one* **7**(6)**:** e38538

Heit JA, Armasu SM, Asmann YW, Cunningham JM, Matsumoto ME, Petterson TM, De Andrade M (2012) A genome-wide association study of venous thromboembolism identifies risk variants in chromosomes 1q24. 2 and 9q. *Journal of Thrombosis and Haemostasis* **10**(8)**:** 1521-1531

Hinds DA, Buil A, Ziemek D, Martinez-Perez A, Malik R, Folkersen L, Germain M, Malarstig A, Brown A, Soria JM, Dichgans M, Bing N, Franco-Cereceda A, Souto JC, Dermitzakis ET, Hamsten A, Worrall BB, Tung JY, Sabater-Lleal M (2016) Genome-wide association analysis of self-reported events in 6135 individuals and 252 827 controls identifies 8 loci associated with thrombosis. *Human molecular genetics* **25**(9)**:** 1867-74

Morange P-E, Bezemer I, Saut N, Bare L, Burgos G, Brocheton J, Durand H, Biron-Andreani C, Schved J-F, Pernod G (2010) A follow-up study of a genome-wide association scan identifies a susceptibility locus for venous thrombosis on chromosome 6p24. 1. *The American Journal of Human Genetics* **86**(4)**:** 592-595

Rühle F, Witten A, Barysenka A, Huge A, Arning A, Heller C, Krümpel A, Mesters R, Franke A, Lieb W (2016) Rare genetic variants in SMAP1, B3GAT2 and RIMS1 contribute to pediatric venous thromboembolism. *Blood*: blood-2016-07-728840
